# Evaluation of inter- and intra-rater reliability of video analysis in ski and snowboard cross

**DOI:** 10.3389/fspor.2026.1746697

**Published:** 2026-04-09

**Authors:** Björn Bruhin, Mara Gander, Matthias Gilgien, Michael Romann

**Affiliations:** 1 Swiss Federal Institute of Sport Magglingen (SFISM), Magglingen, Switzerland; 2 Swiss-Ski, Worblaufen, Switzerland; 3 Department of Physical Performance, Norwegian School of Sport Sciences, Oslo, Norway; 4 Alpine Health and Innovation Foundation, Center of Alpine Sports Biomechanics, Udligenswil, Switzerland; 5 Faculty of Science and Medicine, University of Fribourg, Fribourg, Switzerland

**Keywords:** injury mechanism, winter sports, surrogate measure of injury risk, rater training, video analysis, event categorization

## Abstract

**Introduction:**

Systematic video analysis of events is a widely applied method for examining the mechanisms and underlying causes of sports injuries. Yet, the use of multiple raters poses a considerable challenge, as achieving high inter-rater reliability in video-based assessments is inherently difficult. This study evaluated the inter- and intra-rater reliability of video analysis for identifying events leading to potential injuries in winter sports, focusing on snowboard cross (SBX) and ski cross (SX).

**Method:**

A team of four (4) raters reviewed the video footage. 644 situations were reviewed, categorized by parameters such as crash type, course trajectory, and competitor behaviour. A standardized process was established for training the raters to classify defined situations as Crash (CR), Time of no return (TNR), Rank Shift (RS), Out of balance (OOB), Contact (CT), Avoided Contact (ACT). Inter-rater reliability was assessed using Fleiss’ Kappa and Cronbach’s Alpha, while Cohen’s Kappa was used to evaluate intra-rater reliability.

**Results:**

Categories with distinct, easily identifiable outcomes, such as time of no return and crash, exhibited high inter-rater reliability. Only minor differences exist in the literal interpretation of the values for inter-rater reliability between Cronbach's Alpha and Fleiss’ Kappa. Categories with more nuanced interpretation, such as out-of-balance situations and athlete contact, showed moderate reliability. In contrast, categories like avoided contact showed lower reliability values Intra-rater reliability ranges from fair to moderate across all raters. Clearly identifiable events such as CR and TNR were recognized perfectly, while the other categories show a more ambiguous pattern.

**Conclusion:**

This study advances the field of sports analysis by proposing a standardized methodology for video analysis in sports with high injury incidence, specifically SBX and SX. Categories with very clear definitions of situations were identified with high inter-rater reliability (CR TNR). Others were classified with moderate accuracy across raters (RS, OOB, CT), whereas some categories could not be reliably distinguished (ACT), even following structured training. The same pattern could also be observed in the intra-rater reliability. This method allows for a higher volume of cases to be reliably analysed, which could inform more robust injury prevention strategies.

## 1 Introduction

In winter sports, snowboard cross (SBX) and ski cross (SX) are sports in the FIS (International Ski and Snowboard Federation) World Cup (WC) series since 2002 and have been characterized by a high injury prevalence. SBX shows one of the highest injury incidences at the Winter Olympic Games (1–3). During the 2018 Winter Olympics for instance, 12% of all athletes incurred at least one injury, of which 26% occurred in SBX and 25% in SX events (4). These numbers indicate the need for a more thorough investigation into the injury mechanisms in these events.

Systematic video analysis of injury and potential injury events is a frequently used method for understanding the mechanisms and underlying causes of injuries occurrences in sports. Unlike traditional retrospective methods, such as questionnaires or standardized forms (1, 2, 4), video analysis provides an objective description of time and motion of an event. This approach significantly reduces recall bias and observer subjectivity which are common limitations in other methods of injury investigation (5). Qualitative video analysis has been applied in multiple studies across various sports to dissect the intricacies of potential injury events (6–14). In some cases, video analysis is combined with the use of surrogate measures of injury risk (SMoIR), which are indirect indicators that mitigate the constraints of small sample size (15, 16). Potential parameters for a surrogate measure in alpine sports could include, for example, out-of-balance situations. However, the effectiveness of this approach relies on the assumption that findings in surrogate measures will be translatable into actual injuries.

As such, SBX and SX injury situations have been analyzed using video analysis and specific analysis forms concerning injury-related mechanism and skier behavior (7–9, 12, 14). Furthermore, Randjelovic and colleagues (12) also incorporated additional factors, such as description of the race course with obstacle types (jumps, roller sections etc.), competitor behavior, and a qualitative description of the circumstances leading to the injury in SX races.

In the past, video footage has been analyzed by a primary investigator (9), an author group (12) or experts in the sport (7, 8, 14) to identify time, type of injury, injury mechanism, and cause for the injury. In instances where the assessment was conducted in a group review, the potential injury events were initially evaluated individually, after which a collective assessment was conducted to achieve a unified understanding. To this end, the video recordings were subjected to repeated review until a consensus was reached among the raters (7, 12, 14). However, the involvement of multiple raters represents a significant challenge when analyzing video footage, as achieving high inter-rater reliability is difficult. Subjective judgment and differing levels of expertise among raters can lead to differences in the analysis and interpretation between raters. O’Donoghue (17) discusses these challenges in the context of performance analysis in sports, emphasizing the importance of maintaining reliability in observational data. Furthermore, it is unclear if the same rater consistently assesses the same situation equally over time (intra-rater reliability).

### 1.1 Objectives of this study

Although previous studies have examined the rating of video footage of injury cases, SMoIR, and injury mechanisms in winter sports, none have statistically evaluated the consistency of ratings between independent blinded raters in video analysis of high-performance sports. This study is the first to compare the degree to which individual video raters are comparable in their recognition of situations, using a clear definition, in both cross-sectional and longitudinal analyses. Therefore, this study aimed to develop and validate a standardized video-analysis method that quantifies rating consistency both between different raters and within the same rater over time for identifying potential injury-risk situations in SBX and SX.

## 2 Materials and methods

To assess inter-rater and intra-rater reliability in video analysis a large volume of video footage from SX and SBX WC events in 2020 was collected and assessed. Two experts selected 78.8 hours of television footage and 2390 runs from WC races in SX, and 13.8 hours of television footage and 914 runs from WC races in SBX for this study. Three Ski Cross World Cup competitions (Montafon, Austria; Veysonnaz, Switzerland; Idre Fjäll, Sweden) were selected, with all runs (Training and Competition) recorded at each venue. Additionally, two Snowboard Cross World Cup events (Montafon, Austria; Veysonnaz, Switzerland) with all runs (Training and Competition) were included. The experts defined the different events to be classified from the rater group, created course descriptions, as well as collected and provided the video material. The rater group consisted of four video raters. The four video raters had to identify and categorize predefined events and assign them to the obstacle, respectively segment on a prepared Microsoft Excel file (Version 16.0, Microsoft Corporation, Redmond, WA, USA, 2016).

### 2.1 Video recording

Video recordings of SBX WC races were sourced from Montafon (AUT) and Veysonnaz (SUI), while those of SX WC races were obtained from Innichen (ITA), Idre Fjäll (SWE), Montafon, and Veysonnaz. These recordings were taken by the research crew during the 2018/2019 and 2019/2020 racing seasons using handheld video cameras from various positions along the race course. Additionally, television footage was obtained from Infront Productions (Italy) and archived on Dartfish.tv (Version 8.8.7.0, Dartfish SA, Fribourg, FR, Switzerland, 2021).

### 2.2 Event definition

**Figure 1:**
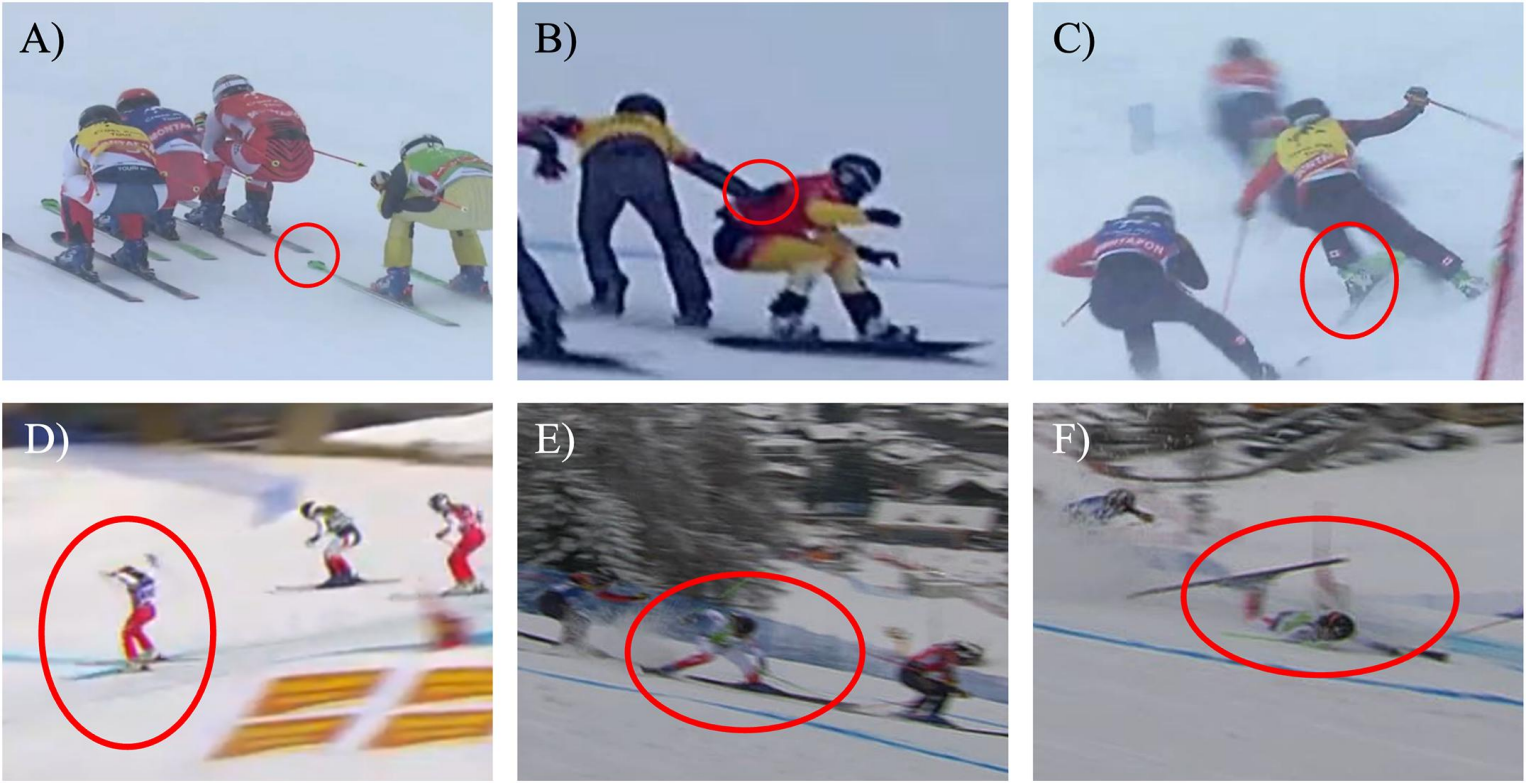
Examples for **(A)** RS, **(B)** CT, **(C)** ACT shown in upper row, from left to right and **(D)** OOB, **(E)** TNR, **(F)** CR, shown in lower row, from left to right.

**Figure 2:**
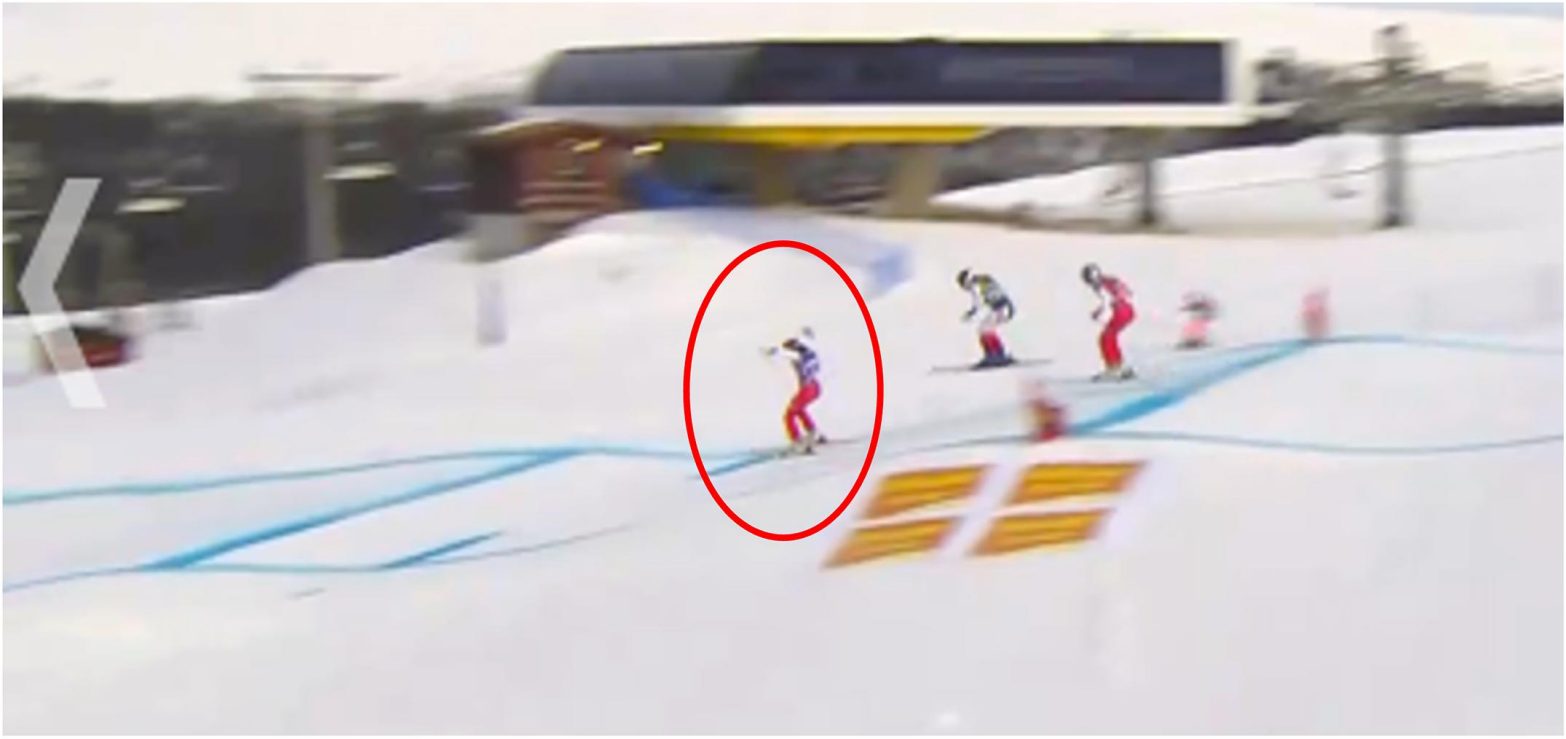
Example of an OOB event definition and a short explanation. Print screen from Dartfish.tv (explanation on this video: “OOB, because skis are not parallel and backward leaning”).

**Figure 3:**
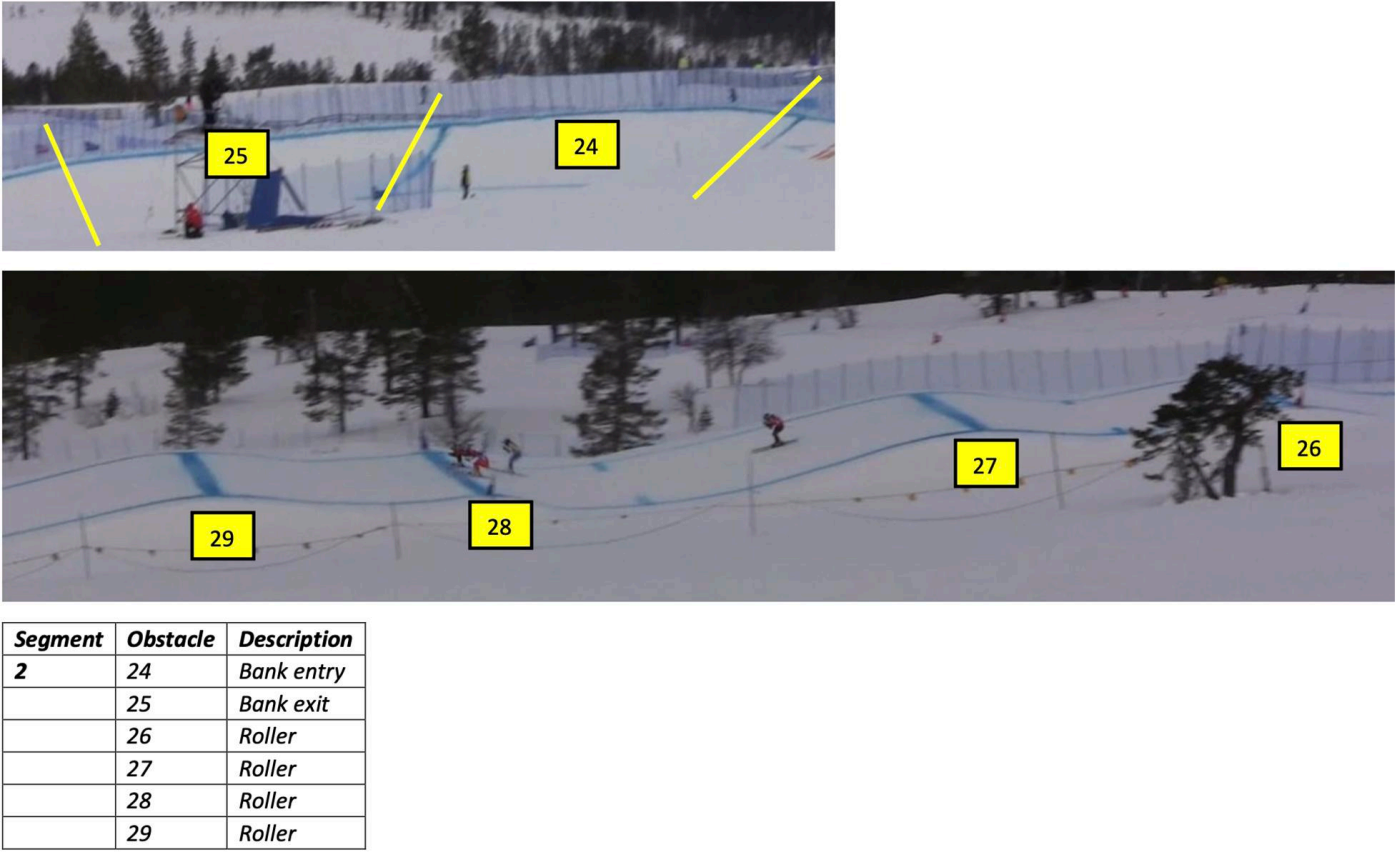
Validation process including days (d) between each step.

The events that were to be identified and classified by the raters were as follows: rank shift (RS), contact (CT), or avoided contact (ACT), out of balance (OOB), time of no return (TNR), and crash (CR). A document *(see* Supplementary Material *for complete documentation)* containing written definitions and pre-rated video clips including short descriptions was provided to the rater group by the two experts (Figures 1, 2). Definitions were elaborated between the experts and the rater group in group sessions during the familiarization phase (Figure 3). Furthermore, the experts shared pre-rated video sequences of events (on Dartfish.tv) with the raters, which outlined which events were to be rated and which were not.

#### 2.2.1 Rank shift (RS)

RS was defined as a position displacement. The opponents’ ski or board tips and ends served as the minimum distance between two athletes. If a competitor caught up with another one (same ski/board boot level), it was recorded as a RS, too.

#### 2.2.2 Contact and avoided contact (CT)

CT was defined as a contact of any part of the skier/snowboarder with an opponent (pole to pole contact excluded). Avoided contact (ACT) was defined as an actively avoided contact by one athlete. For example, by breaking or lifting one ski over the opponent's ski without contact. The athlete was required to be in immediate proximity and to have attempted to evade an impending contact with another athlete.

#### 2.2.3 Out of balance and out of balance linked to contact (OOB)

In general, unintentional change of direction of the skis/board, unintentional movement and out of control were indicators for OOB. It was specified between obstacle categories. Back weighted, unintentional arm movements, upright upper body, and skies/board not parallel to the ground were indicators of OOB at jumps. Inward leaning, outside arm and ski in the air indicated an OOB situation during direction changes. The athlete had three options, (1) either extra effort to recover from the OOB situation by shaking hastily with their arms or to support themselves by contact to opponent (OOB linked CT) or terrain, (2) or TNR.

An OOB linked CT occurs when an athlete is in an OOB situation and, in response to a loss of balance, unintentionally contacts an opponent.

#### 2.2.4 Time of no return (TNR)

A TNR situation was mandatory before a CR. A potential criterion was an OOB situation before the TNR, which resulted in an unavoidable CR.

#### 2.2.5 Crash (CR)

CR was defined as a full body contact with the snow. A TNR was mandatory for a CR.

### 2.3 Event localisation

**Figure 4:**
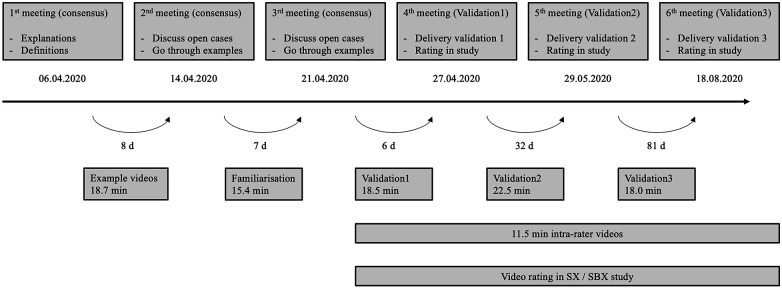
Print screen from one track information sheet. Including the number of the obstacles in these pictures highlighted bold.

Documents containing the track information for all WC races were provided for the purpose of event localization *(see* Supplementary Material *for complete documentation)*. Every obstacle along each course was depicted, numbered, and labelled (Figure 4). The obstacles were classified into three main categories: jumps, rollers, and direction changes (18). Within the categories, obstacles were further classified according to the different types of jumps, direction change (bank, negative, giant slalom gate, corner, roller turn) and rollers (single, double, triple). Jumps were classified according to their take-off and landing areas. Direction changes were subdivided into two categories: entry and exit. Furthermore, obstacles were delineated into three levels of detail: (a) going up the obstacle, (b) going down the obstacle and (c) in between (after) obstacle(s).

### 2.4 Rating of potential injury events

The analysis team consisted of four raters. The raters were students, each of whom held a bachelor's degree or higher education in sports science or sport physiotherapy, with no prior experience in video rating. All raters had a background in winter sports. The extent of their involvement in SX and SBX varied. The video footage was uploaded to Dartfish.tv in.mp4 format. The video material was analysed using Dartfish.tv due to its capabilities for slow-motion and frame-by-frame playback. Eighteen video sequences, with a total duration of 54.6 min, were selected from the entire video material of the races and cropped by an expert (Table 1). The reliability of the method was evaluated in a progressive manner, commencing with a familiarization phase and concluding with three subsequent validations.

**Table 1:** Summary of the number of runs across the different phases of the validation process.

Category	Familiarization	Validation 1	Validation 2	Validation 3	Intra-rater
Rater [*n*]	4	4	4	4	4
Video sequence duration [min]	15.4	18.5	22.5	18.0	11.5
Total heats [*n*]	6	6	6	6	4
Heats SBX [*n*]	2	2	2	2	2
Heats SX [*n*]	4	4	4	4	2
Heats female [*n*]	3	3	3	4	3
Heats male [*n*]	3	3	3	2	1

Video recordings were subjected to detailed examination with the objective of identifying and localizing specific events by human raters. The experts developed a specific analysis form in Microsoft Excel. The form included a variety of information, such as the video name on dartfish.tv, the gender of the participant, the type of heat, time, segment number, obstacle number, and level of detail for each obstacle. Additionally, it included the ranking, event category, and bib number, as well as the order in the start gate. Raters marked each event in a new row with the number “1” and added the bib number(s).

The term “potential events” refers to instances, during which the raters concurred on the absence of an event, regardless of its specific category. Consequently, these events were not documented in the protocol (19). Nevertheless, the raters were required to determine whether an event had occurred or not. One expert was responsible for rating all potential events in each validation video sequence. As an example for the OOB category, all situations in which a rater questioned whether an event should be classified as an OOB situation were identified and coded by an expert. To compute Fleiss’ Kappa correctly, it is necessary to define the set of potential events. The calculation of agreement then considers both all events in which at least one rater has identified a situation, and the potential situation in which every rater explicitly made a decision not to classify the situation, as the reference population.

### 2.5 Evaluation process

The experts in the field of SBX and SX were providing supervision for the rater group. Each rater was required to rate the video sequences independently from the other raters on the provided analysis form. Figure 3 delineates the evaluation process, which spanned a period of four months. As recommended in the literature, the raters underwent training and the extent to which they rated the same events was measured incrementally (20, 21). As an initial step, raters were introduced to the task in an online group session and completed the familiarization phase by rating six (6) SBX and SX heats. The raters underwent the following training regime: after each stage, one expert reviewed the protocols. Events, where consensus between raters was low, and unclear events, where raters were unsure, were discussed and clarified in online group sessions. Each meeting lasted approximately two hours. Prior to the meetings, inconsistencies were identified and specifically prepared by the experts. The raters were able to share their perspectives, and these were discussed within the group. If consensus was reached, this decision was adopted. In challenging cases, the experts provided and explained the final decision.

Another aim of the study was to assess the intra-rater reliability. For this purpose, Validation3 included two (2) video sequences from validation1 and two (2) from validation2, thereby allowing repeated evaluations.

### 2.6 Statistics

For each validation, all events identified by the four raters, were collated into a single Excel document by one expert. Events were deemed to be identical if they were reported within a one-second window. The one-second window was selected for events that may extend over a certain time interval. TNR can represent such a case. The athlete is in the process of falling, but there is some temporal ambiguity regarding the exact moment of event occurrence; however, it is clear that the event does take place. The inter-rater reliability was evaluated using Fleiss’ Kappa (30) and Cronbach's Alpha (23), both of which range from 0 to 1. The use of Fleiss’ Kappa and Cronbach's Alpha is appropriate, as the capture complementary aspects of reliability. While Fleiss’ Kappa quantifies the degree of agreement among multiple raters beyond chance, Cronbach's Alpha evaluates the internal consistency of the ratings. Both parameters were employed to examine differences and similarities across the results. Fleiss’ Kappa was calculated by taking the difference between the observed agreement (P̅) and the agreement expected by chance (P̅_e_) $\bar{P}-{\bar{P}}_{e}$, and dividing it by the maximum possible agreement beyond chance $1-\;{\bar{P}}_{e}$. The interpretation of Fleiss’ Kappa values is as follows: values of 0 indicate poor agreement, 0.2 indicates slight agreement, 0.4 indicates fair agreement, 0.6 indicates moderate agreement, 0.8 indicates substantial agreement, and 1.0 indicates almost perfect agreement (22). Cronbach's Alpha was used to assess internal consistency, specifically, how well a set of items (in this case, rater judgments) measures a single construct. The interpretation of Cronbach's Alpha values is as follows: 0.5 is considered unacceptable, 0.6 is poor, 0.7 is questionable, 0.8 is acceptable, 0.9 is good, and 1.0 is excellent (23). Although Cronbach's Alpha is commonly used for continuous data and internal consistency, it can also be applied in the context of inter- or intra-rater reliability to evaluate consistency across multiple raters or repeated measures (24). In this study, Cronbach's Alpha was employed as an approximate indicator of internal consistency among raters. Cohen's kappa was employed to measure intra-rater reliability and is interpreted similarly to Fleiss’ kappa. It focusses on the agreement of a single rater's evaluations over time beyond what is expected by chance (31).

As a summary, different reliability coefficients address complementary aspects of measurement quality: Fleiss’ Kappa is particularly useful for assessing inter-rater reliability across multiple raters, while Cronbach's Alpha provides insights into the internal consistency of the ratings, and Cohen's Kappa is well-suited for evaluating the consistency of repeated measures by the same rater (25).

## 3 Results

### 3.1 Inter-rater reliability

In total, 644 events were recorded, comprising 270 events of validation1, 192 instances of validation2, and 182 instances of validation3. Table 2 presents the mean Fleiss’ Kappa and Cronbach's Alpha values for each event across all three validations. The Fleiss’ Kappa coefficients for TNR and CR were nearly perfect. The results for RS, CT, and OOB were deemed to be satisfactory, whereas those for ACT were inconclusive, and OOB linked to CT in an unsatisfactory manner. Cronbach's Alpha for TNR and CR demonstrated excellent reliability. RS and CT were deemed to be satisfactory, whereas OOB was deemed to be questionable. Both ACT and OOB-linked CT were deemed to be unacceptable.

**Table 2:** Fleiss’ Kappa and Cronbach's Alpha per event type.

Category	RS	CT	ACT	OOB	OOB linked CT	TNR	CR
Validation 1 - number of events [*n*]	89	38	14	114	11	2	2
Validation 2 - number of events [*n*]	61	35	10	79	3	2	2
Validation 3 - number of events [*n*]	63	26	11	68	4	5	5
Total (Validation 1, 2, 3) - number of events [*n*]	213	99	35	261	18	9	9
Fleiss’ Kappa [*κ*]	0.50	0.51	0.22	0.45	0.10	1.00	1.00
	Fair	Fair	Slight	Fair	Poor	Substantial	Substantial
Cronbach's Alpha [*α*]	0.81	0.81	0.55	0.79	0.20	1.00	1.00
	Acceptable	Acceptable	Questionable	Questionable	Unacceptable	Excellent	Excellent

RS, rank shift; CT, contact; ACT, avoided contact; OOB, out of balance; OOB linked CT, out of balance linked to contact; TNR, time of no return; CR, crash.

**Figure 5:**
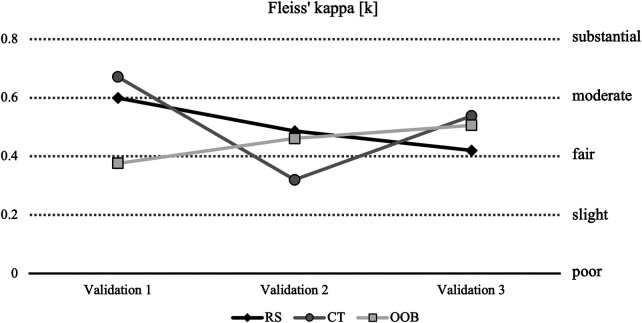
Fleiss’ Kappa for RS, CT and OOB. RS, rank shift; CT, contact; OOB, out of balance.

Fleiss’ Kappa in RS decreased from moderate to fair (*κ*_V1_ = 0.60; *κ*_V2_ = 0.49; *κ*_V3_ = 0.42) (Figure 5). CT decreased from moderate to slight and increased to fair (*κ*_V1_ = 0.67; *κ*_V2_ = 0.32; *κ*_V3_ = 0.54). OOB increased from slight to fair (*κ*_V1_ = 0.38; *κ*_V2_ = 0.46 ; *κ*_V3_ = 0.51). Fleiss’ Kappa for TNR and CR were almost perfect along each validation (*κ*_TNR_ = 1.00; *κ*_CR_ = 1.00). Fleiss’ Kappa for ACT increased from slight (*κ*_V1_ = 0.13; *κ*_V2_ = 0.01) to moderate (*κ*_V3_ = 0.51). Fleiss’ Kappa for OOB-linked CT increased from unacceptable (*κ*_V1_ = -0.18; *κ*_V2_ = -0.03) to moderate (*κ*_V3_ = 0.50). The between-validation fluctuations observed for CT and RS likely reflect an iterative calibration effect induced by the feedback sessions between validations.

**Figure 6:**
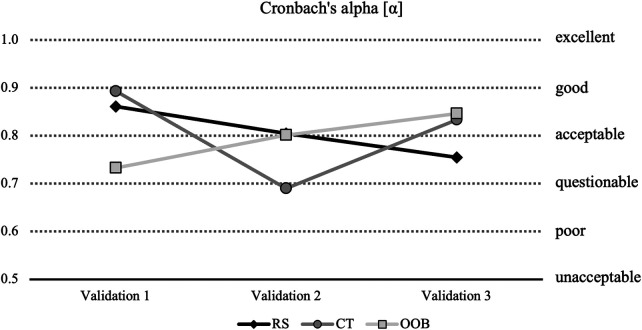
Cronbach's Alpha for RS, CT and OOB. RS, rank shift; CT, contact; OOB, out of balance.

Cronbach's Alpha in RS decreased from acceptable to questionable (*α*_V1_ = 0.86; *α*_V2_ = 0.80; *α*_V3_ = 0.75) (Figure 6). CT decreased from good to poor and increased to acceptable (*α*_V1_ = 0.89; *α*_V2_ = 0.69; *α*_V3_ = 0.83). OOB increased from questionable to acceptable (*α*_V1_ = 0.73; *α*_V2_ = 0.80; *α*_V3_ = 0.85). Cronbach's Alpha for TNR and CR were excellent for each validation (*α*_TNR_ = 1.00; *α*_CR_ = 1.00). Cronbach's Alpha for ACT increased from unacceptable (*α*_V1_ = 0.44; *α*_V2_ = 0.40) to good (*α*_V3_ = 0.81). Cronbach's Alpha for OOB-linked CT increased from unacceptable (*α*_V1_ = -0.40; *α*_V2_ = 0.33) to questionable (*α*_V3_ = 0.67).

### 3.2 Intra-rater reliability

**Figure 7:**
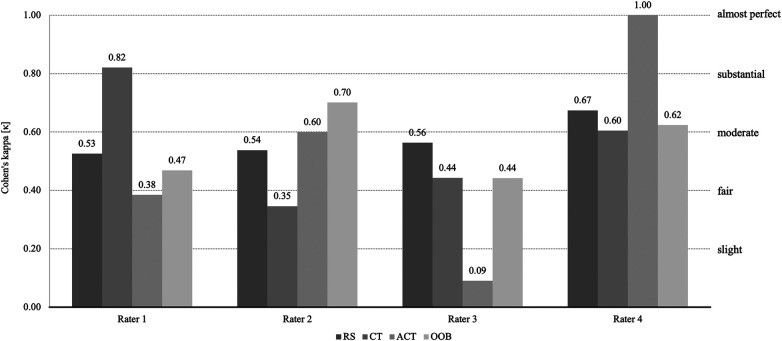
Cohen's Kappa for intra-rater reliability.

A total of 137 events were included in the assessment of intra-rater reliability (45 RS, 17 CT, 8 ACT, 59 OOB, 4 OOB linked CT, 2 TNR, 2 CR). The mean values across all event categories for the individual raters are comparable. TNR and CR were identified perfectly. For the other categories, ambiguous results were observed between the individual raters. Intra-rater reliability differed between raters. Cohen's Kappa coefficients for RS, CT, ACT and OOB ranged from slight to almost perfect (Figure 7): RS (*κ*_R1_ = 0.53; *κ*_R2_ = 0.54; *κ*_R3_ = 0.56; *κ*_R4_ = 0.67), CT (*κ*_R1_ = 0.82; *κ*_R2_ = 0.35; *κ*_R3_ = 0.44; *κ*_R4_ = 0.60), ACT (*κ*_R1_ = 0.38; *κ*_R2_ = 0.60; *κ*_R3_ = 0.09; *κ*_R4_ = 1.00), OOB (*κ*_R1_ = 0.47; *κ*_R2_ = 0.70; *κ*_R3_ = 0.44; *κ*_R4_ = 0.62).

The average of all four (4) raters Cohen's Kappa, of the events were similar (*κ*_RS_ = 0.58; *κ*_CT_ = 0.55; *κ*_ACT_ = 0.52; *κ*_OOB_ = 0.56), except for TNR and CR (*κ*_TNR_ = 1.00; *κ*_CR_ = 1.00). Differences emerged in the mean rater coefficients, when considering the individual raters, (*κ*_R1_ = 0.70; *κ*_R2_ = 0.70; *κ*_R3_ = 0.59; *κ*_R4_ = 0.82).

## 4 Discussion

This Study aimed to assess the inter- and intra-rater reliability of video analysis in SX and SBX events. The results demonstrate that inter- and intra-rater reliability varied significantly across the different event categories. Fleiss’ Kappa and Cronbach's Alpha indicated almost perfect reliability for the TNR and CR categories, underscoring the consistency with which these events were identified and classified by the raters. In contrast, RS, CT, ACT, and OOB showed lower inter- and intra-rater reliability, highlighting the challenges associated with standardization of objective event description criteria.

Events, such as TNR and CR are characterized by distinct and easily recognizable situations. This results in their clear and objective definitions, which likely facilitated consistent identification and made them less susceptible to varying interpretations. The lower reliability in the RS, CT, ACT, and OOB categories suggests that these events are more complex and subjective in nature. For instance, interpreting whether an athlete was OOB or whether a CT was intentional or unintentional involves nuanced judgement, which can vary between raters (12). The variability in these categories may also reflect the complexity of interactions and movements in SBX and SX races, as multiple factors influence the rating of potential injury events, such as camera perspective, weather conditions, or many athletes beside each other.

In this setting, a highly comparable pattern can be observed between Fleiss’ Kappa and Cronbach's Alpha. It is noteworthy that, in this dataset, differences occur in the calculated values, but there is little difference in either their interpretation (Table 2) or their substantive meaning. Moreover, previous studies on injury mechanisms in performance winter sports also reported challenges regarding inter-rater reliability for subjective assessments (7–9, 12). In most cases, a consensus meeting effectively resolved the issue (7–12, 14). However, the consensus approach dramatically limits the number of cases that were assessed in previous studies ((7) = 19 cases; (8) = 20 cases; (9) = 69 cases; (12) = 33 cases; (14) = 57 cases). This reduction of cases is necessary because each case must be discussed in plenary to reach a consensus. In this validation study, it was possible to examine 644 situations. However, it should be noted as a limitation that these were not only crashes as reviewed in the abovementioned studies (Table 2). To allow such procedures in elite sports, detailed definitions of what constitutes each event are essential (26).

In order to achieve a high level of agreement, the raters were provided with the training program (Figure 3), since it is well established that training includes reviewing pre-rated video sequences and clarifying questions to events in consensus meetings can improve the consistency of evaluations, and thus, enhance inter-rater reliability (27). Furthermore, intra-rater reliability also showed significant variation across event categories and raters, which may indicate challenges in maintaining consistency over time. This can be explained by possible shifts in rater attention, and evolving interpretations. These findings align with Shrout and Fleiss (28) that highlight the difficulties of achieving high intra-rater reliability in complex observational tasks. Over time, a rater's criteria for evaluating events may shift, leading to judgment drift, which is the change in a rater's interpretation of event categories between assessments. This is often due to subtle, unconscious changes in the way they apply the rating criteria (29). Furthermore, learning effects may occur as raters become more experienced with the task (25). Another possible explanation for the large variations in the intra-rater results is the number of events per category (see Table 2). In categories with a high number of events, the results varied substantially less.

The underlying methodology (video-based event coding, predefined categories, temporal windows, rater training, consensus procedures, and reliability assessment) is applicable across sports. Video analysis represents an established tool in numerous sport disciplines, particularly in contexts where complex and dynamic situations must be captured objectively. Sports characterized by the following features are especially likely to benefit from direct transferability: high velocity, interactions between athletes, fall and collision events, and clearly identifiable movement phases. While the system itself is transferable, specific categories (e.g., type of contact, fall mechanisms, positional changes) may require sport-specific adaptations. The demonstrated inter- and intra-rater reliability indicates that the system can be applied consistently. This constitutes a central prerequisite for transferability, as a reliable system is, in principle, reproducible in other contexts. For each new sport, the category set should be adapted, raters should be trained, and reliability should be reassessed.

### 4.1 Limitations

A main limitation of this study is the reliance on human judgment, which is inherently subjective and prone to error and variability. Potential cognitive fatigue and judgment drift over time may have affected intra-rater reliability, particularly in complex and nuanced event categories. Therefore, future research should aim to address the main limitations identified in this study by reducing subjectivity in video analysis. This could involve the development of a software that can assist or complement human raters. Raters were able to complete the program individually and were instructed to do so only while maintaining a very high level of concentration. However, because the ratings were conducted in a decentralized manner, the execution could not be controlled.

Refining event definitions and training protocols could further improve reliability, especially in more subjective event categories. Some categories (TNR, CR, OOB linked CT) contained a small number of evaluated events (<20); therefore, the findings for these categories should be interpretated with caution.

## 5 Conclusion

In Conclusion, this study represents an important step towards improving the systematic video analysis in performance sports with high injury risks like SBX and SX. Even though the methodology utilized in this study requires further refinement, it is applicable for video analysis in SBX and SX. The assessment of agreement by Fleiss’ Kappa and Cronbach's Alpha constitutes a valid and complementary approach for evaluating inter-rater reliability. Additionally, Cohen's Kappa, as an index of intra-rater reliability, can be considered valid considering these results. The sufficient inter-rater agreement in key event categories supports further video analysis and practical applications in SBX and SX. The improvement in intra-rater reliability observed throughout the training process underlines the effectiveness of structured training programs and group meetings for enhancing rater consistency. The presented system allows, in the future, a large number of video sequences to be evaluated by multiple raters. Given the finding that inter-rater agreement is acceptable, substantially larger datasets can be assessed within a shorter time frame. This also opens the possibility of identifying hotspots across different courses where various situations occur. The insights gained from this study contribute to a more accurate and reliable framework for analyzing video footage of events leading to potential injuries in SBX and SX, paving the way for better preventative strategies and improved athlete safety in sports with a high injury risk

## Data Availability

The raw data supporting the conclusions of this article will be made available by the authors, without undue reservation.
