# Supplementary material for: Evaluation of inter- and intra-rater reliability of video analysis in ski and snowboard cross
Source: Front Sports Act Living. 2026 Apr 9;8:1746697. doi: 10.3389/fspor.2026.1746697 (PMC13102760; doi:10.3389/fspor.2026.1746697)

## *Definitions of major events for video analysis*

The video is analyzed to localize where on the course events such as contact, out of balance, time of no return and crash happen. Six different event categories and one pending event will be identified:

**Contact (CT)**

What is a contact?

- A contact of any part of the skier/snowboarder (including ski/board and poles) with an opponent
    - Excluding contacts pole to pole of two athletes which do not cause a harmful interference between the athletes

**Avoided Contact (ACT)**

What is an avoided contact?

- An actively avoided contact by one athlete
    - If one athlete reacts quickly to avoid contact by lifting the ski over an opponent's ski or breaking.

**CT** and **ACT** -> If yes:

- Description of the segment and obstacle at which the contact/avoided contact occurs
- Record of the first contact between two athletes
    - if there are more than one contact per obstacle
    - Per obstacle only the first point of contact is marked.

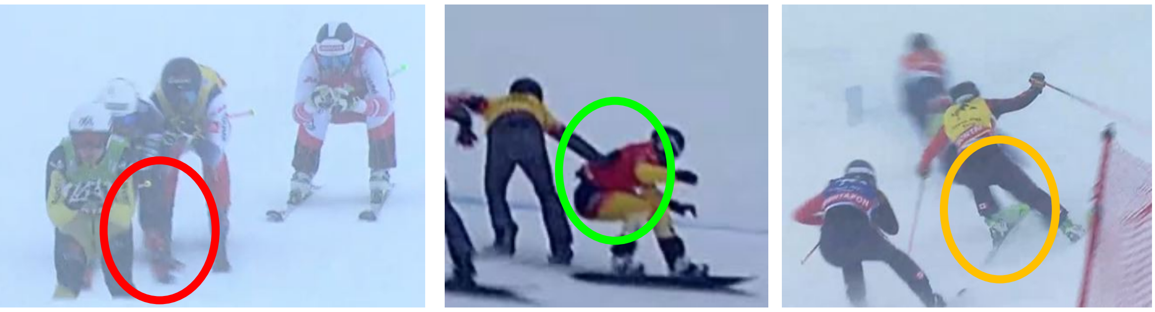

Fig.1 contact in SX (left, marked with red circle, orange for almost contact, avoided by one athlete) and SBX (right, marked with green circle)

**Out of balance (OOB)**

The spot where the cause of loss of balance occurred—the first moment where the skier loses his balance. OOB situations occur at different obstacles and therefore appear in different manner:

Jump

- Back weighted
- Unintentional arm movements
- Upper body upright
- Skis/board are not parallel to the ground

Turn

- Inward leaning

    - Outside arm in the air
    - Outside ski in the air

General

    - Unintentional change of direction of skis/board
    - Unintentionally movement
    - Out of control

Athlete has two options from here,

- Extra effort to recover from this situation f.e.
    - by shaking hastily with arms (upper body) to get back in fore-aft
    - support himself by opponent sideways (CT) or terrain
- Time of no return

**OOB** -> If yes:

- Description of the segment (obstacle) at which the OOB occurs
- Record of the start time of the point of OOB

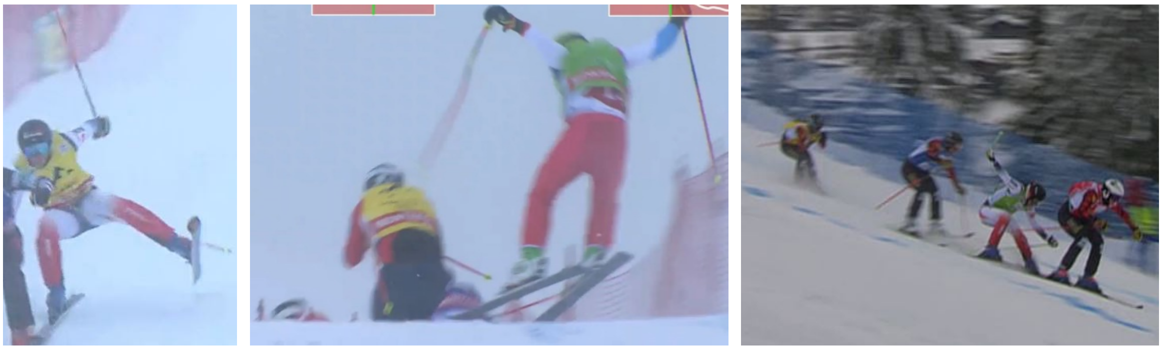

Fig.2 examples of oob situations

**Time of no return (TNR)**

The spot where the fall was unavoidable. It may be identical to or later than the OOB. Only defined for athletes who fell (yes or no).

**TNR** -> If yes:

- Description of the segment (obstacle) at which the tnr occurs
- Record of the start time of the point of tnr

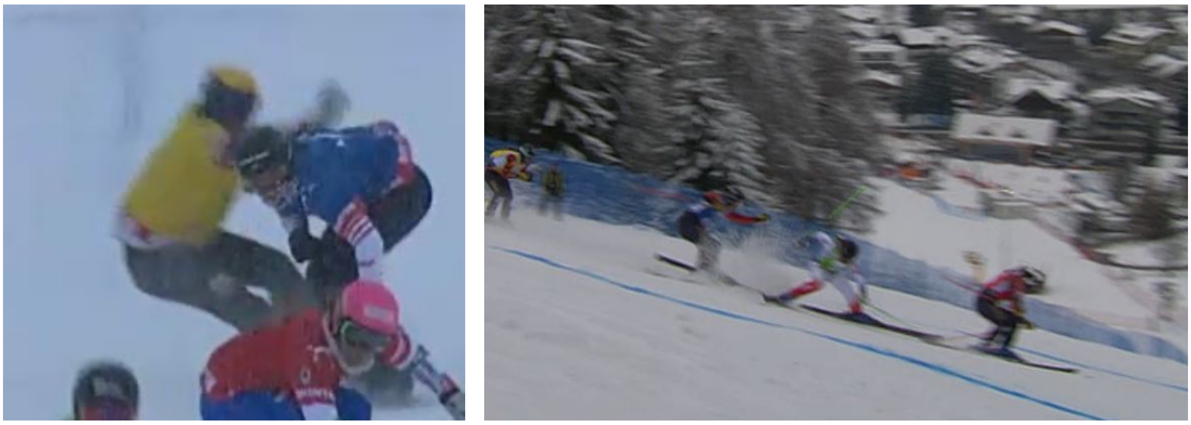

Fig.3 examples of tnr situations

**Crash (CR)**

- Record of the time point of the actual crash

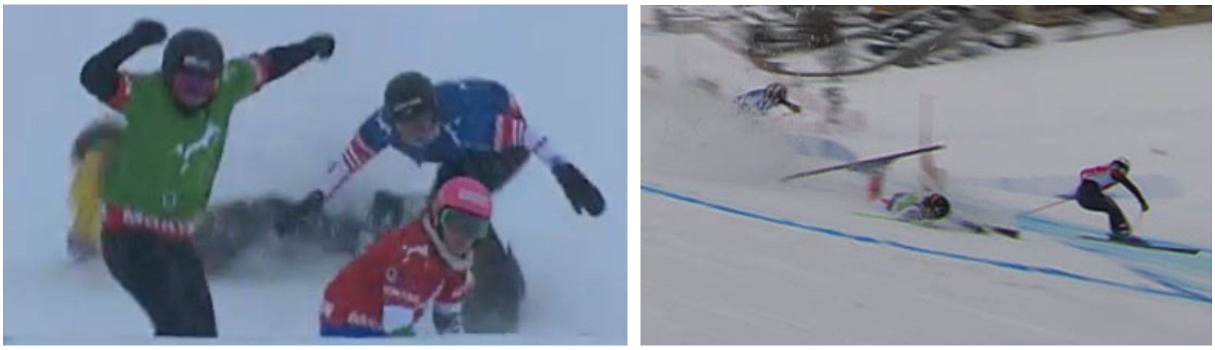

Fig.4 examples of CR situations

**Rank shift (RS)**

- Record of each position displacement
- As a measure, one ski length serves as the minimum distance between two competitors. If two competitors catch up with each other (same level), this is also recorded as a rank shift.
- If there is a situation when one athlete is one ski length ahead of the las athlete, this is a rs, the ranking will then be 1 – 1 – 1 – 4 , Ranks will be reported as the actual rank of the athlete and not only the order, means if there are 3 athletes in rank 1, the fourth athlete is at rank 4 even his rank order is 2.
- For the finish there is no rank shift
  - No writing of the finish from any athlete
  - No rank shifts need to be noted after finish line
  - Important Events (oob, ch, tnr) need to be noted after finish line

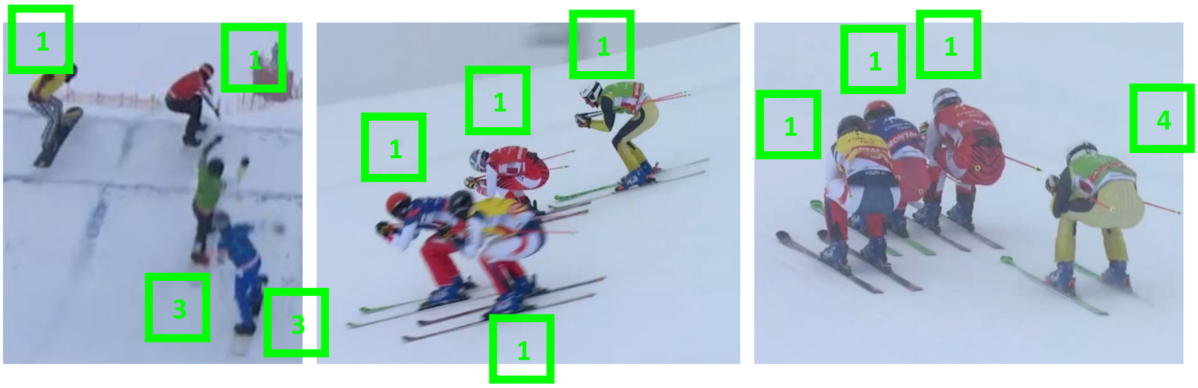

Fig.5 examples of rs situations

Each incidence when athletes touch each other, crash or are injured (identified from the video) is located on the digital terrain model and on trajectories. Local terrain characteristics and biomechanical characteristics (speed, turn radius, forces, etc.) are derived to quantitatively characterize course characteristics and athletes' biomechanics at the locations where athletes interfere, crash or become injured. Importantly, we will be able to record the total number of runs per day. With this information, the events (contact, oob, tnr, crash and rs) can be displayed as normalized in relation to the total runs. Locations with high risk incidences are identified as so-called 'hot spots'.

**Locations**

The definitions of the spots where an event occurred has one more level of detail:

- Going up the obstacle (a)
    - Roller, Jump before the gradient increases significantly, turns marked with color
- Going down the obstacle (b)
    - Roller, Jump not available, turns marked with color
- In between obstacles (after) (c)
    - After an obstacle before the start of the next one.
    - Number is according to the previous obstacle

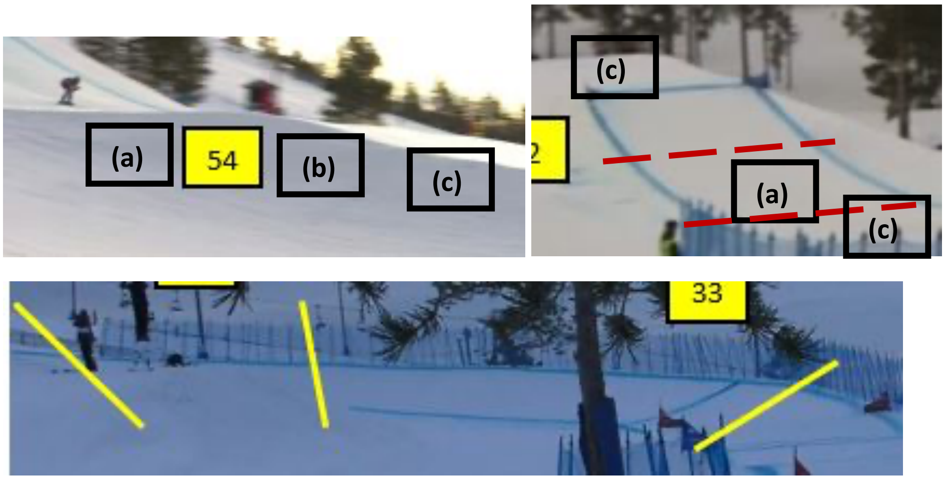

Supplement: Supplementary file 1 [file Datasheet1.pdf]
