# Supplementary material for: Evaluation of inter- and intra-rater reliability of video analysis in ski and snowboard cross
Source: Front Sports Act Living. 2026 Apr 9;8:1746697. doi: 10.3389/fspor.2026.1746697 (PMC13102760; doi:10.3389/fspor.2026.1746697)
Supplement: Supplementary file 2 [file Datasheet2.pdf]

__Montafon Track Information__

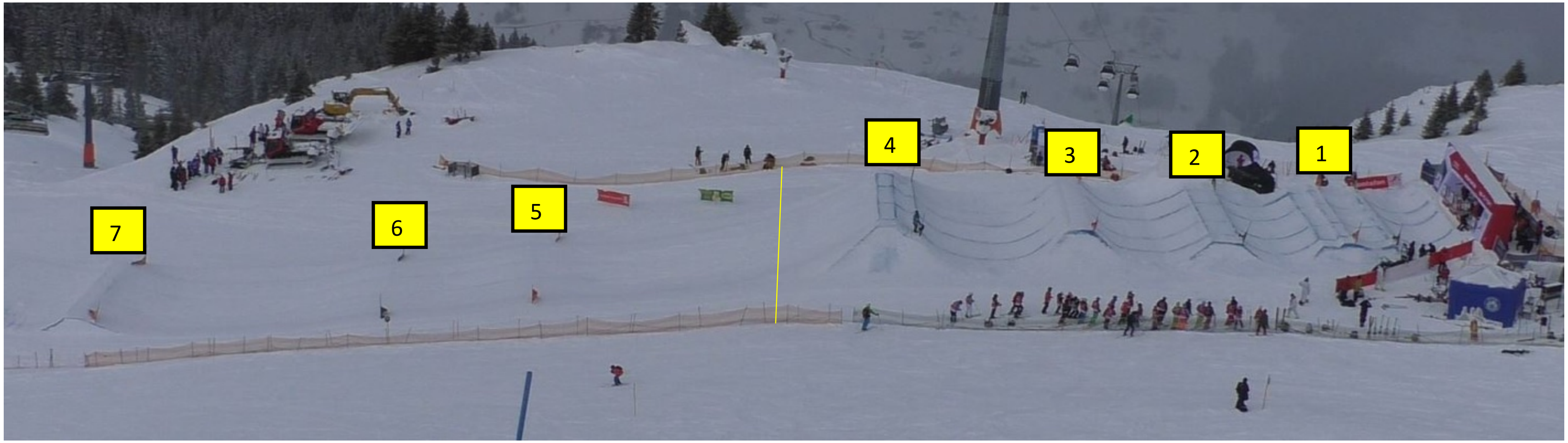

__Segment__	*__Obstacle__*	*__Distance (m)__*	*__Description__*
__1__	*1*	*7- 13*	*Bathtube*
	2	*17- 21*	*Wu tang 1*
	3	*29-31*	*Wu tang 2*
	4	*41-45*	*Wu tang 3*
__2__	*5*	*69*	*Roller smooth 1*
	6	*82*	*Roller smooth 2*
	7	*100*	*Major Jump1 takeoff*

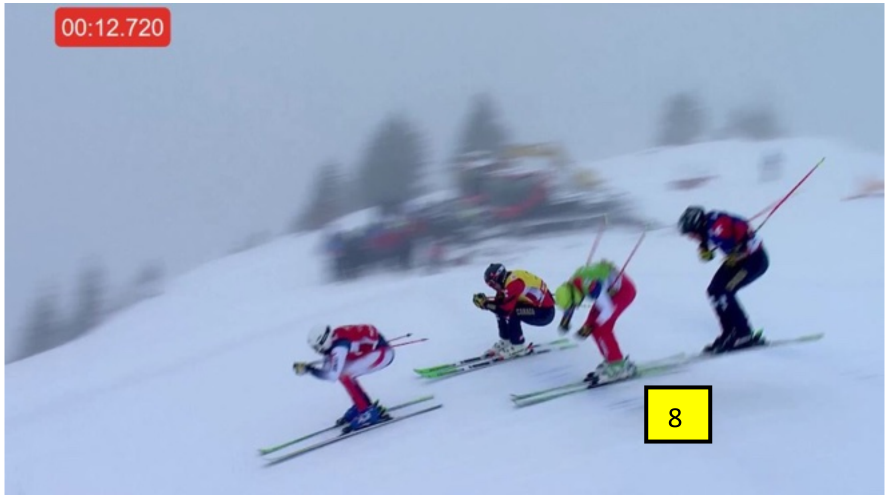

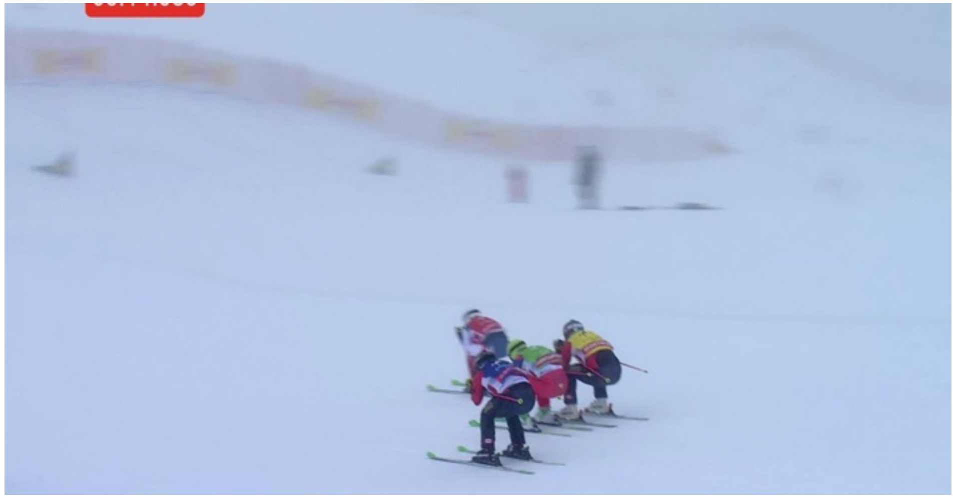

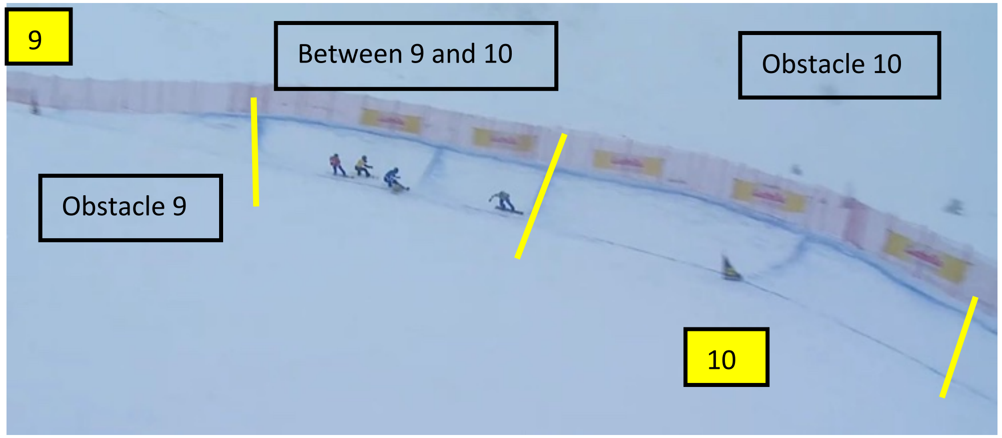

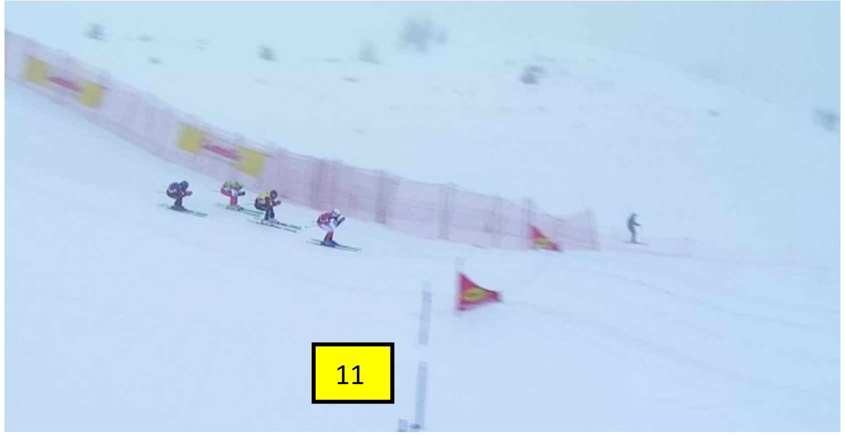

	8	*109*	*Major Jump1 Landing*
3	*9*	*161*	*Turn1 entry*
	10	*202*	*Turn1 exit*
	11	*226*	*Roller, smooth 3*

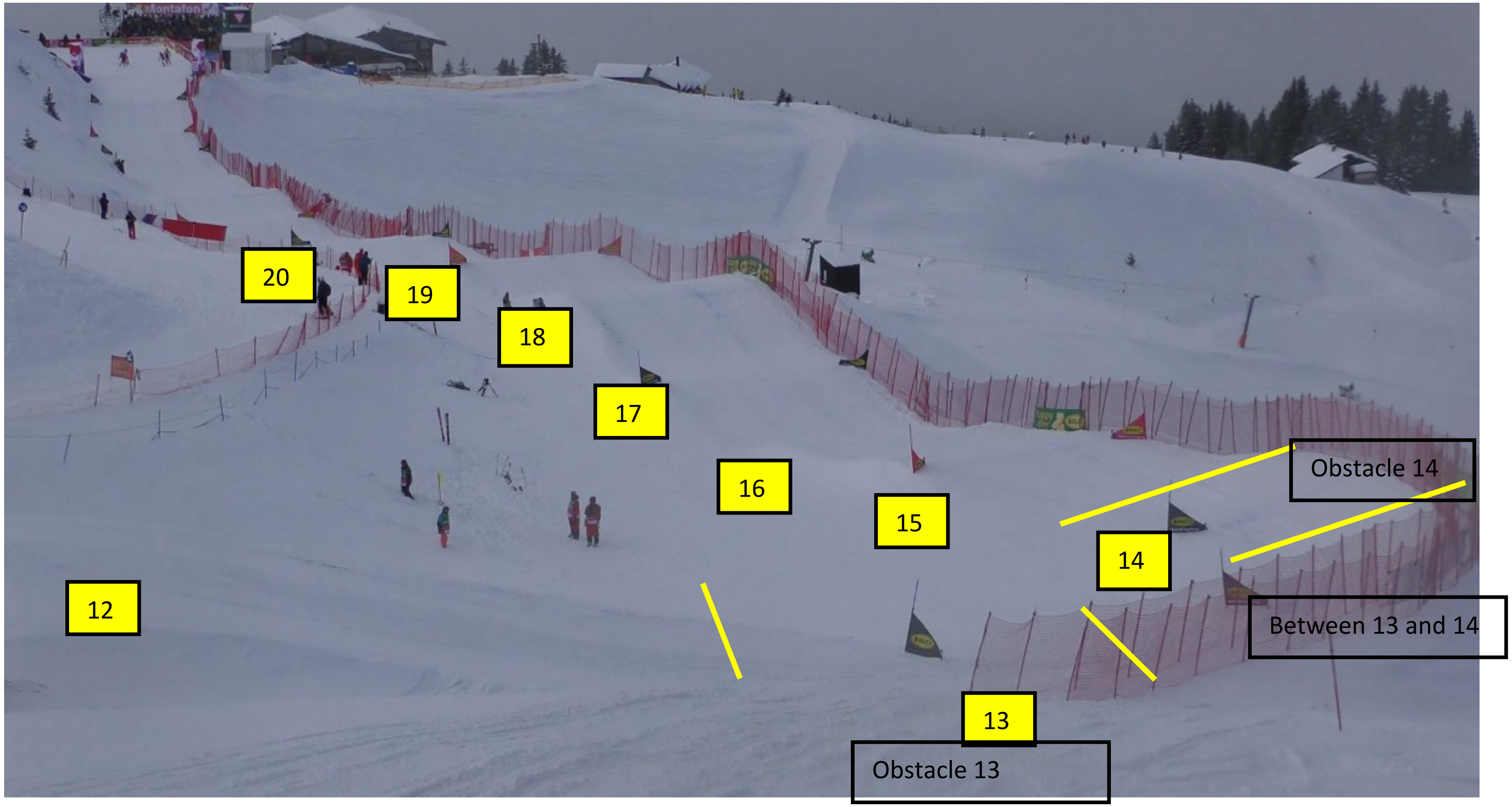

	12	*241*	*Roller, smooth 4*
	13	*262*	*Turn2 entry*
	14	*307*	*Turn2 exit*
4	*15*	*311*	*Roller, smooth 5*
	16	*322*	*Roller, smooth 6*
	17	*338*	*Dragon up*
	18	*354*	*Dragon peak*
	19	*375*	*Dragon down*

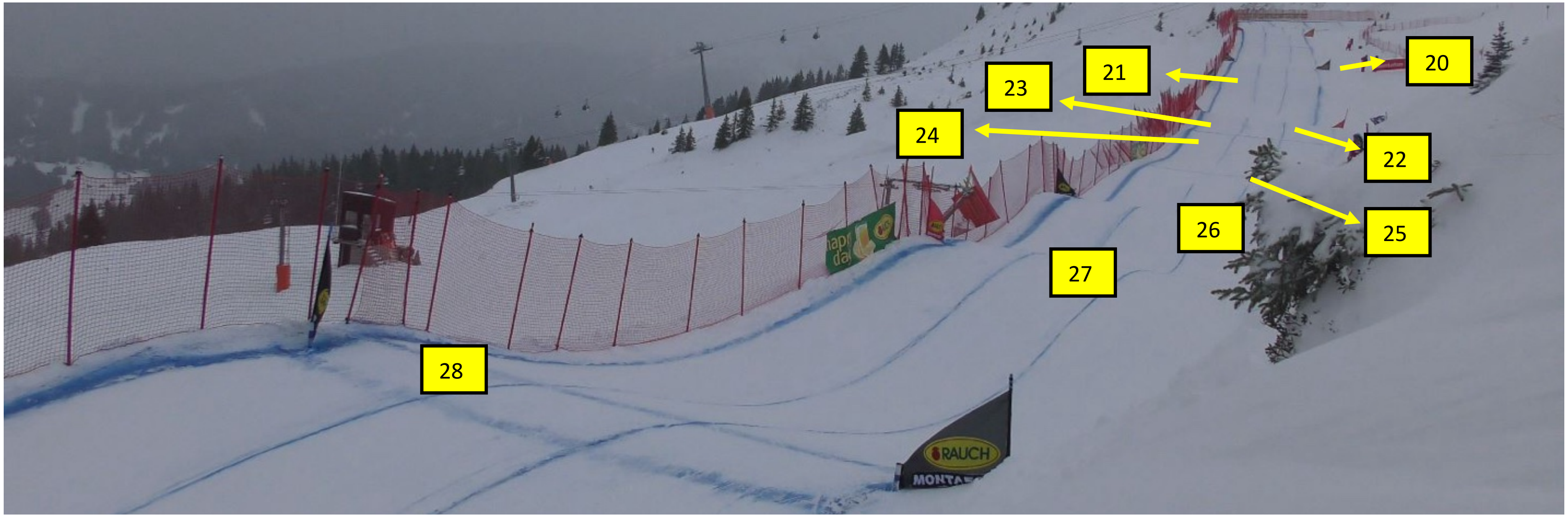

Segment	*Obstacle*	*Distance (m)*	*Description*
	20	*412*	*Roller, smooth 7*
	21	*425*	*Roller, smooth 8*
	22	*457*	*Roller, smooth 9*
	23	*468*	*Roller, smooth 10*
	24	*478*	*Roller, smooth 11*
	25	*496*	*Roller, smooth 12*
	26	*516*	*Roller, smooth 13*
	27	*531*	*Roller, smooth 14*
	28	*556*	*Roller, smooth 15*
	29	*585*	*Finish line*

Segment	*Obstacle*	*Distance (m)*	*Description*
1	*1*	*7- 13*	*Bathtube*
	2	*17- 21*	*Wu tang 1*
	3	*29-31*	*Wu tang 2*
	4	*41-45*	*Wu tang 3*
2	*5*	*69*	*Roller smooth 1*
	6	*82*	*Roller smooth 2*
	7	*100*	*Major Jump1 takeoff*
	8	*109*	*Major Jump1 Landing*
3	*9*	*161*	*Turn1 entry*
	10	*202*	*Turn1 exit*
	11	*226*	*Roller, smooth 3*
	12	*241*	*Roller, smooth 4*
	13	*262*	*Turn2 entry*
	14	*307*	*Turn2 exit*
4	*15*	*311*	*Roller, smooth 5*
	16	*322*	*Roller, smooth 6*
	17	*338*	*Dragon up*
	18	*354*	*Dragon peak*
	19	*375*	*Dragon down*
	20	*412*	*Roller, smooth 7*
	21	*425*	*Roller, smooth 8*
	22	*457*	*Roller, smooth 9*
	23	*468*	*Roller, smooth 10*
	24	*478*	*Roller, smooth 11*
	25	*496*	*Roller, smooth 12*
	26	*516*	*Roller, smooth 13*
	27	*531*	*Roller, smooth 14*
	28	*556*	*Roller, smooth 15*
	29	*585*	*Finish line*

Veysonnaz Track Informations

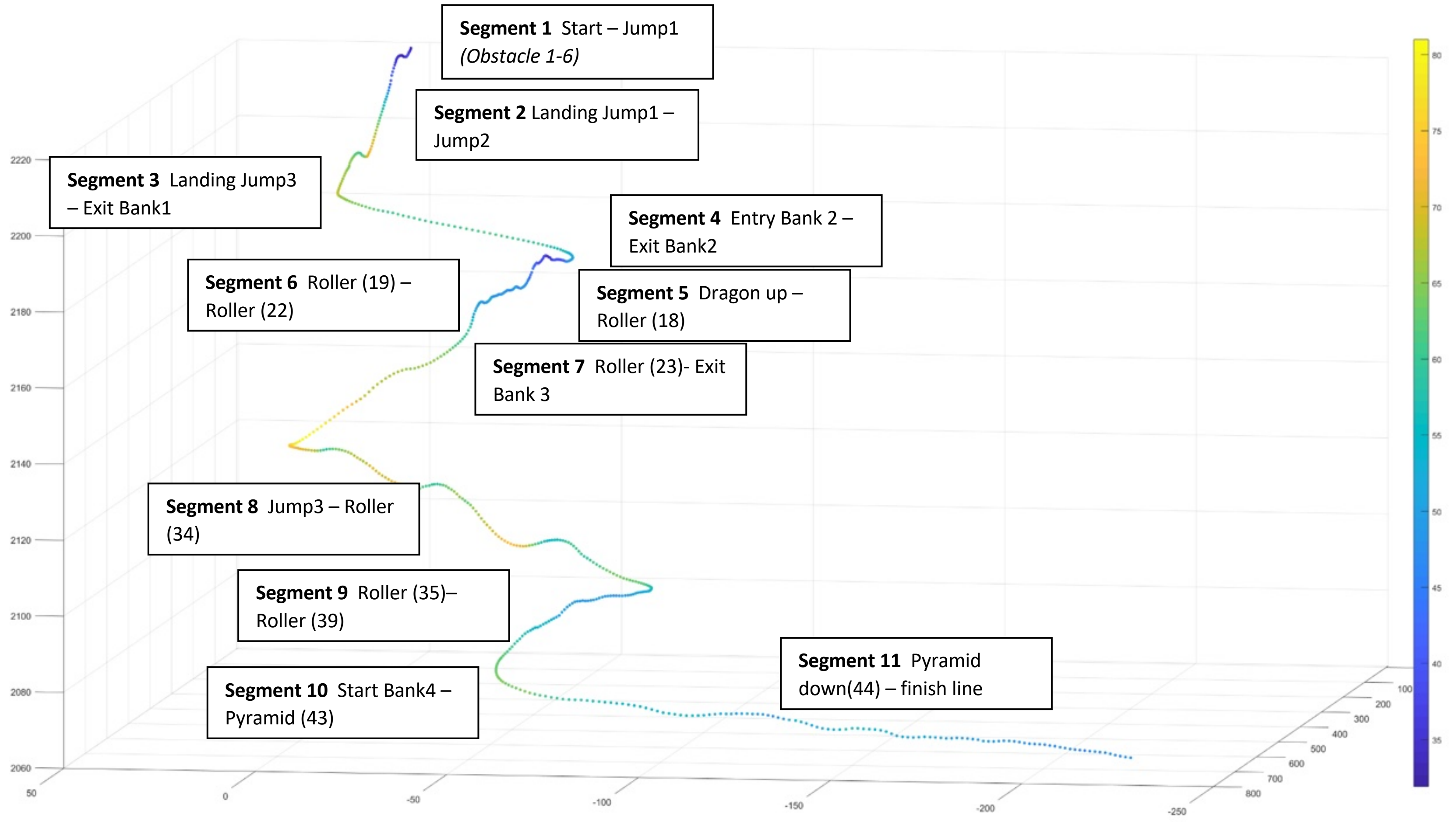

Segment	***Picture***		***Description***
	If a competitor crashes or have a situation 'out of balance'. It will be noticed in which segment and on (or between which obstacles) the situation occurred.<br>F.e. 'Out of balance' segment 1 – 2m before obstacle 6 (major jump1)		
1	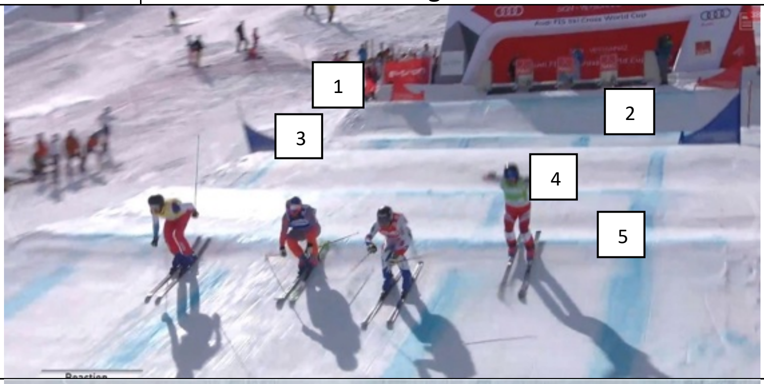		1= Starttable<br>2= Startdrop<br>3= Wu tang 1<br>4= Bathub entry<br>5= Bathub exit<br>6= Major jump1
2	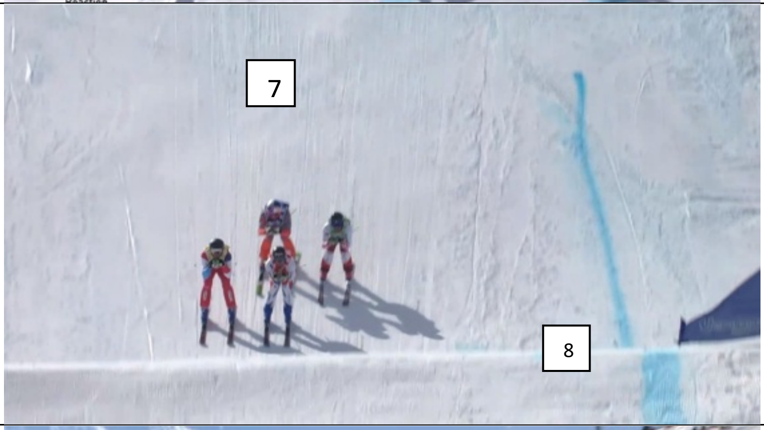		7= Landing<br>8= Major jump2
3	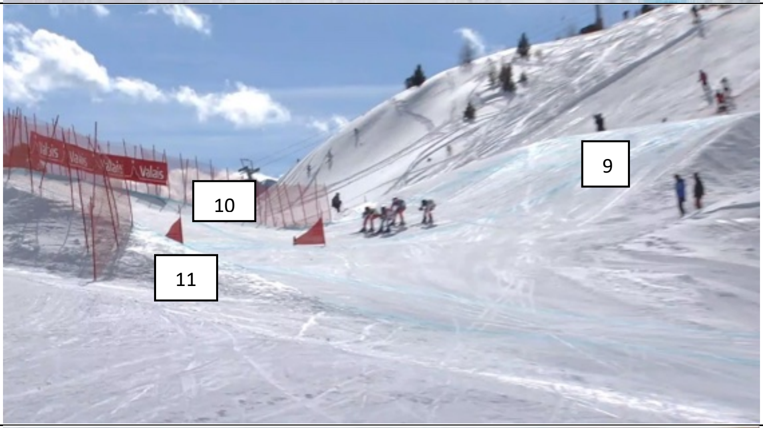		9= Landing<br>10= Bank 1 entry<br>11= Bank 1 exit
4	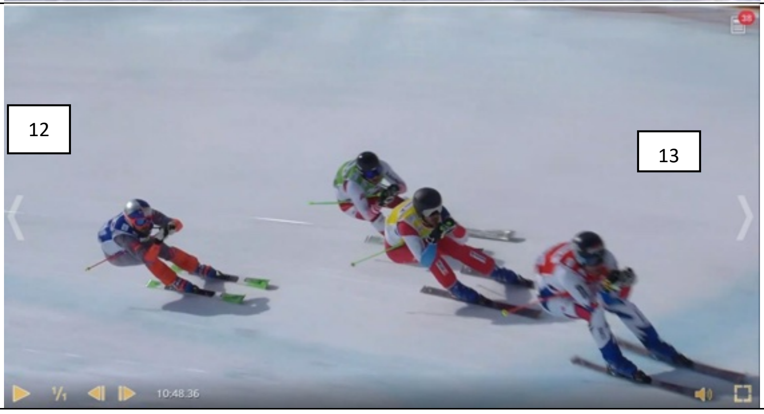		12= Bank 2 entry<br>13= Bank 2 exit<br>(Exit is marked with the blue line)

5	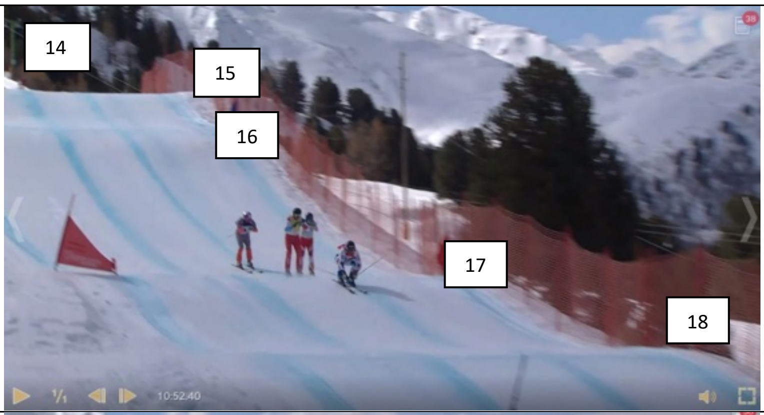		14= Dragon up<br>15= Dragon peak<br>16= Dragon down<br>17= Roller<br>18= Roller
6	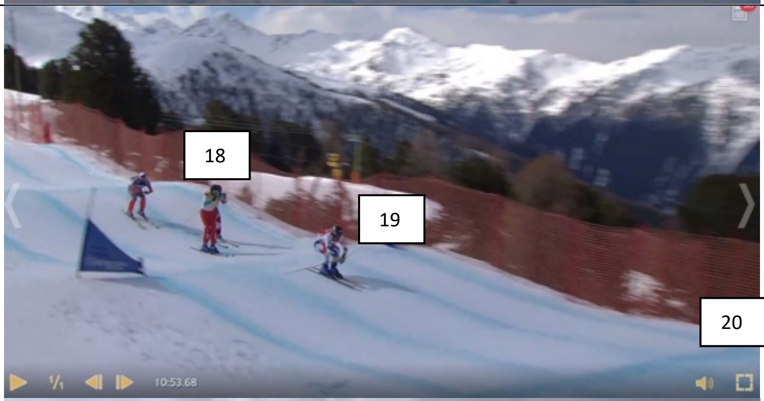 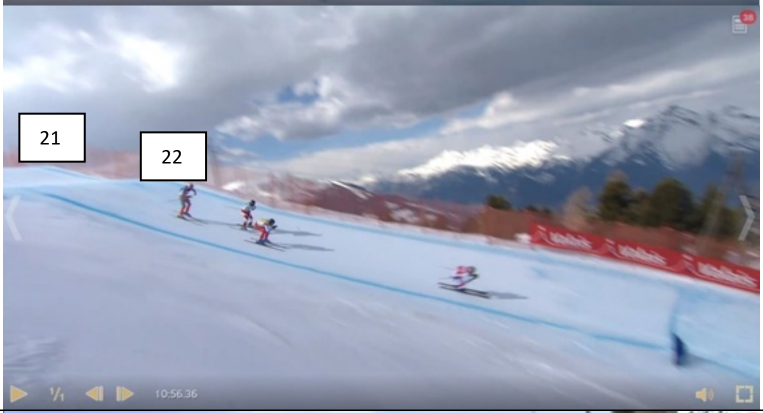		19= Roller<br>20= Roller<br>21= Roller<br>22= Roller
7	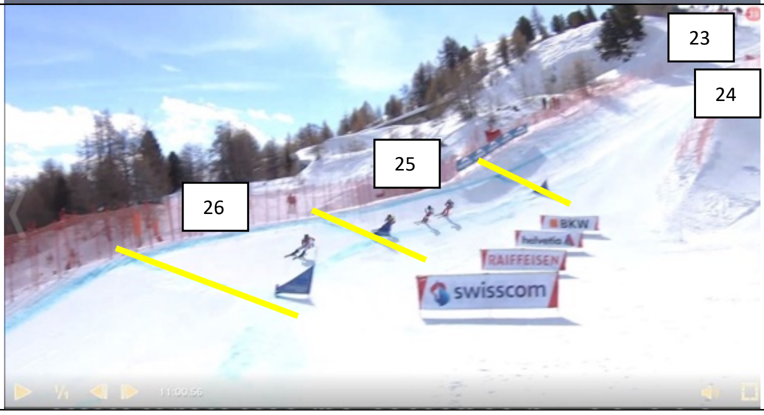		23= Roller<br>24= Roller<br>25= Bank 3 entry<br>26= Bank 3 exit

	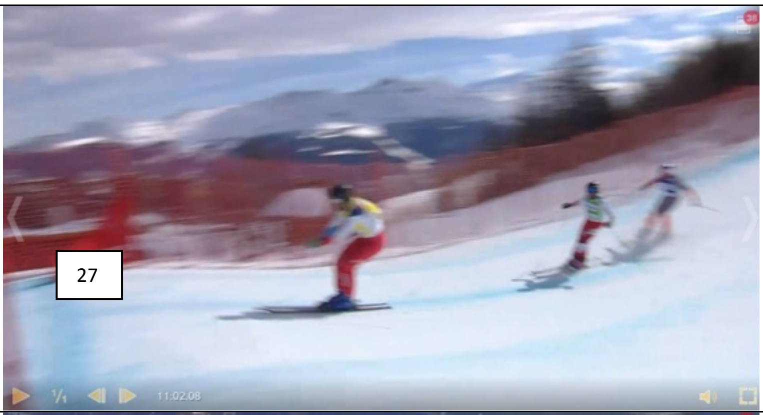		
8	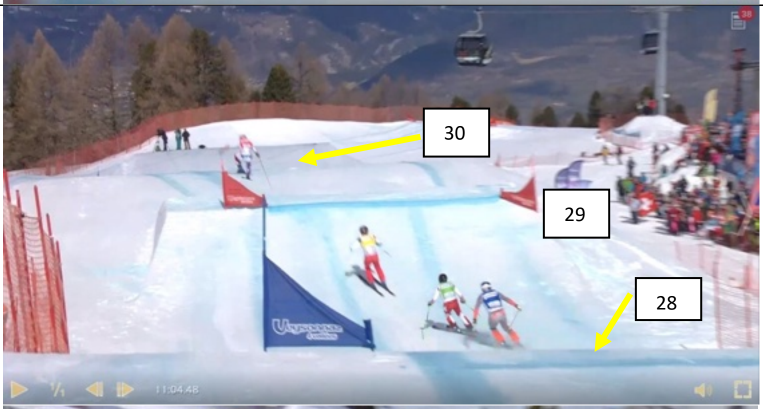<br>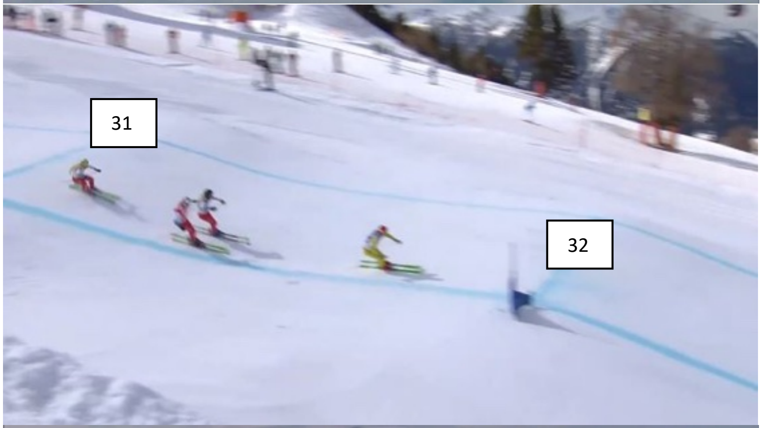<br>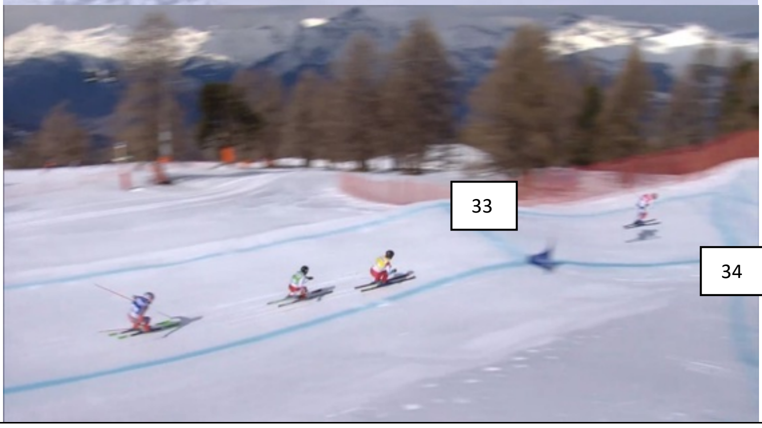		**SX:**<br>27= Major jump 3<br>28= Landing<br>29= Major jump 4<br>30= Landing<br><br>31= Roller<br>32= Roller<br>33= Roller<br>34= Roller

9	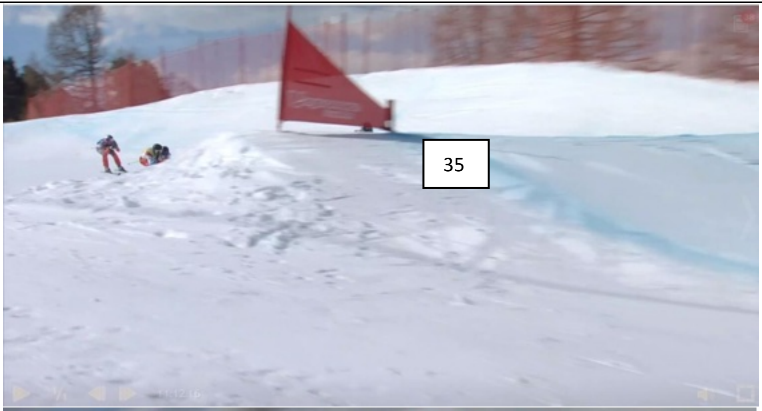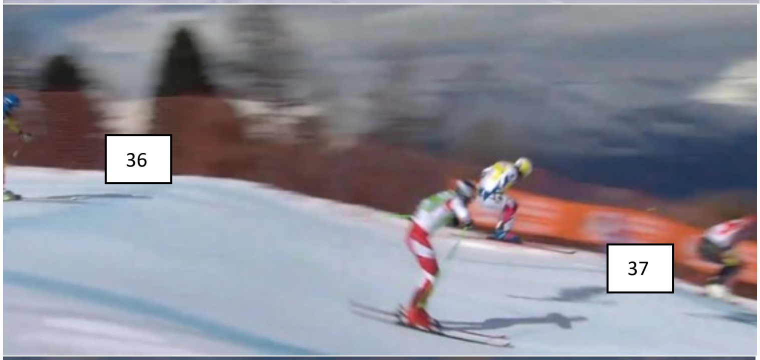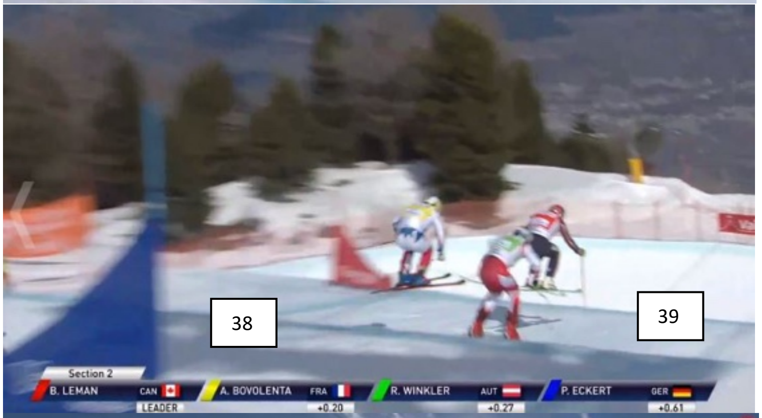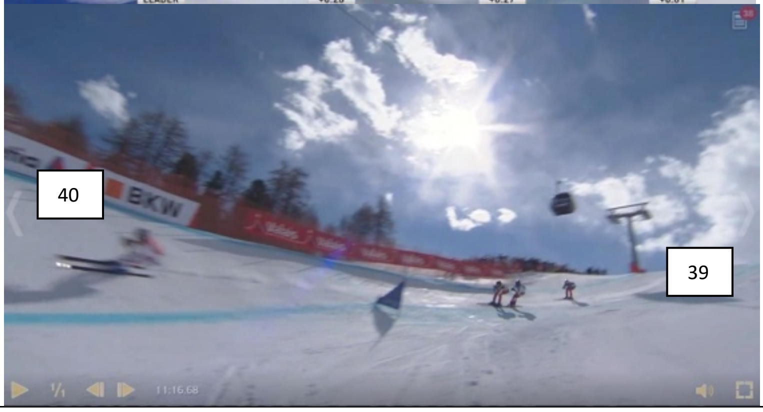		35= Roller<br>36= Roller<br>37= Roller<br>38= Roller<br>39= Roller

10	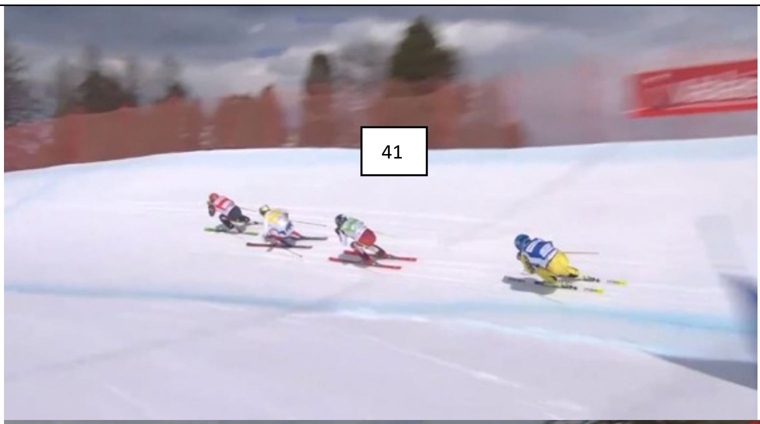 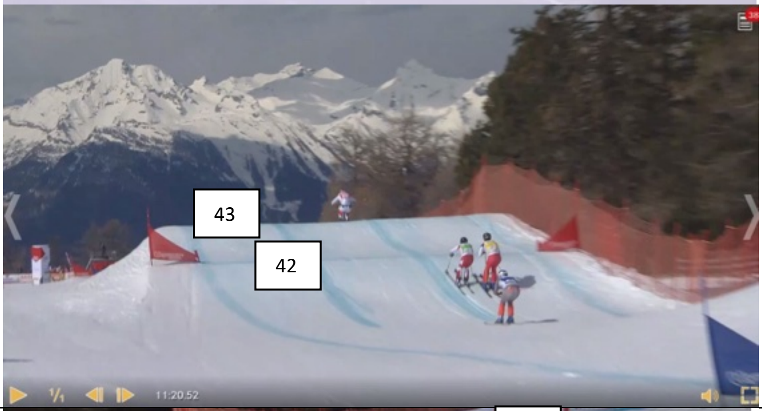		40= Bank 4 entry<br>41= Bank 4 exit<br>42= Pyramid up<br>43= Pyramid<br><br>At the blue line of Roller (43), Segment 41 ends
11	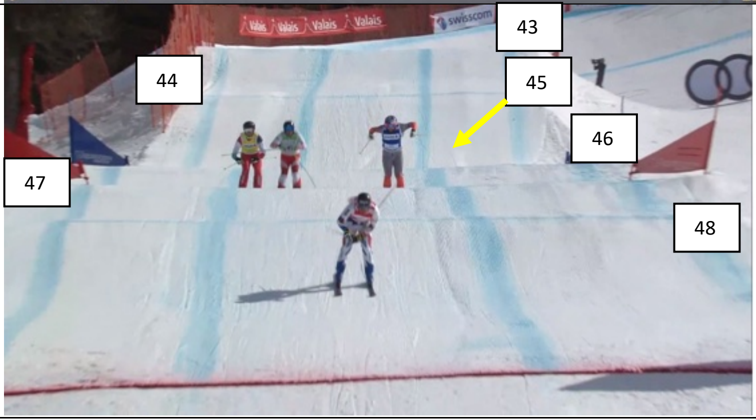		44= Pyramid down<br>45= Roller<br>46= Roller<br>47= Roller<br>48= Roller<br>49= Finish line

Veysonnaz Track Informations

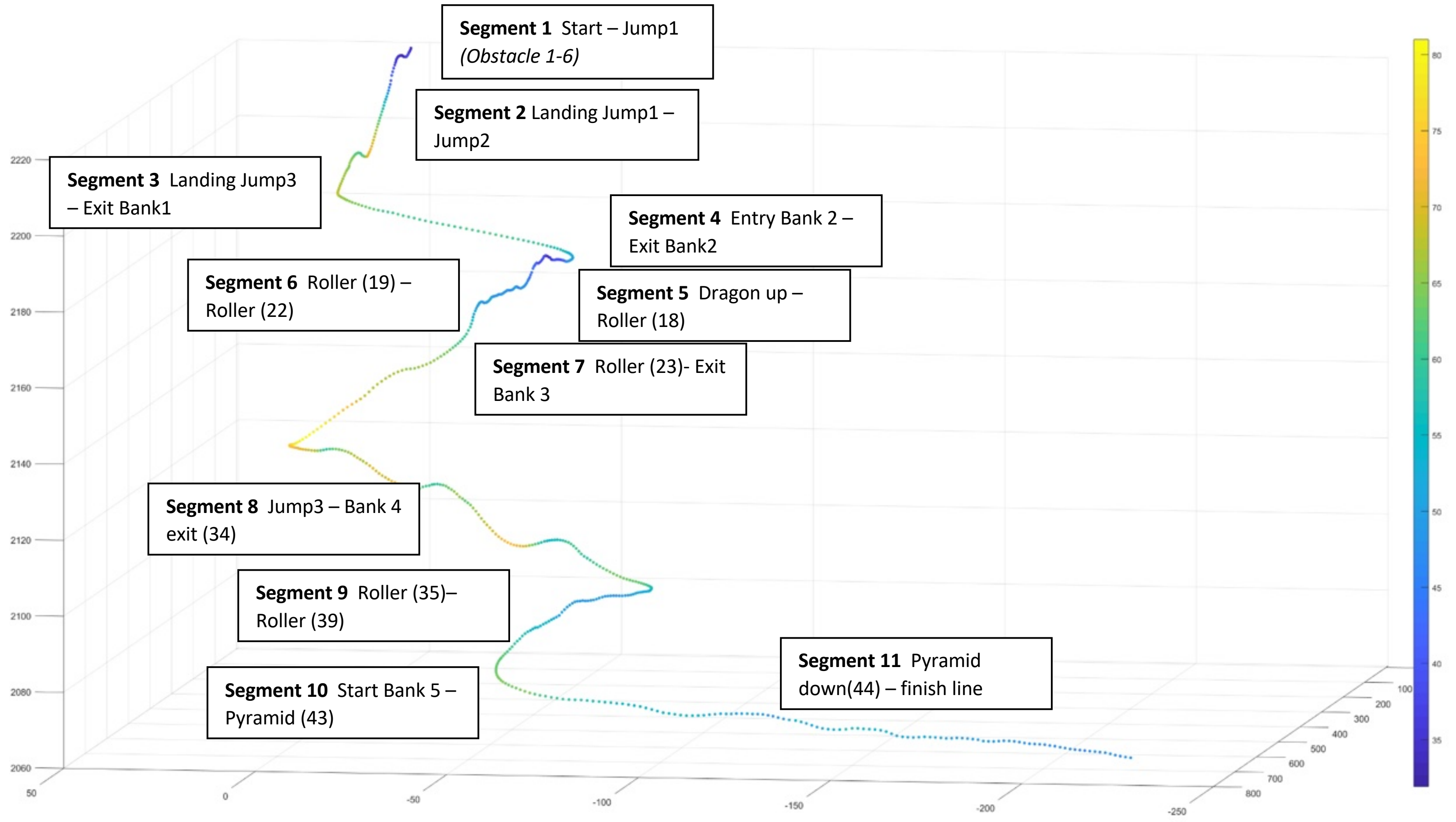

bjoern.bruhin@swiss-ski.ch

Segment	***Picture***		***Description***
	If a competitor crashes or have a situation 'out of balance'. It will be noticed in which segment and on (or between which obstacles) the situation occurred.<br>F.e. 'Out of balance' segment 1 – 2m before obstacle 6 (major jump1)		
1	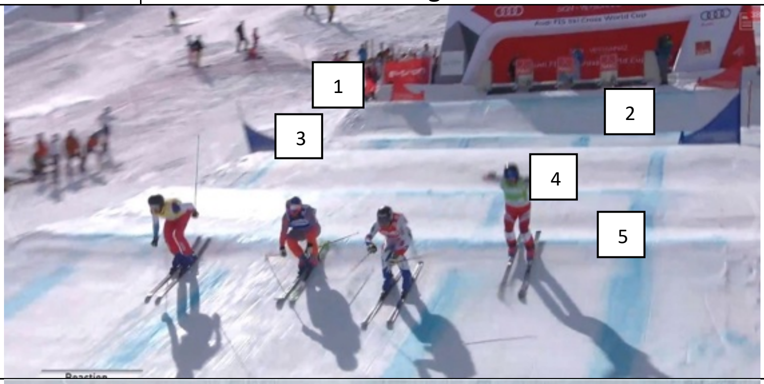		1= Starttable<br>2= Startdrop<br>3= Wu tang 1<br>4= Bathub entry<br>5= Bathub exit<br>6= Major jump1
2	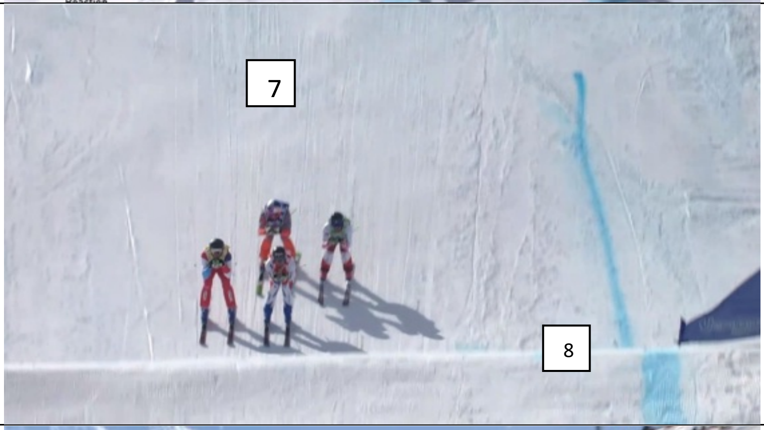		7= Landing<br>8= Major jump2
3	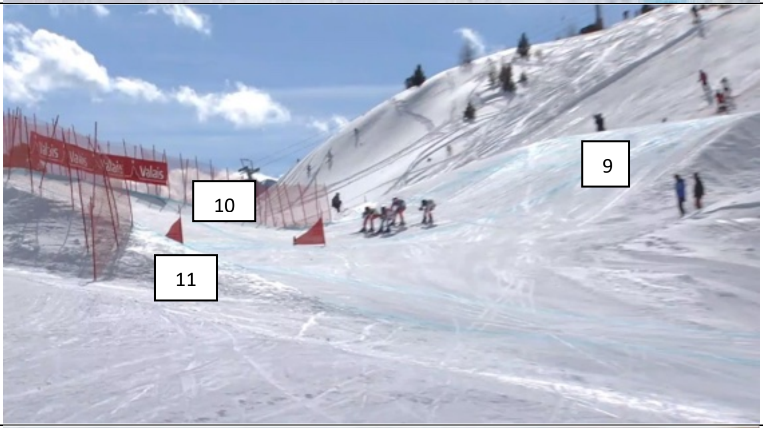		9= Landing<br>10= Bank 1 entry<br>11= Bank 1 exit
4	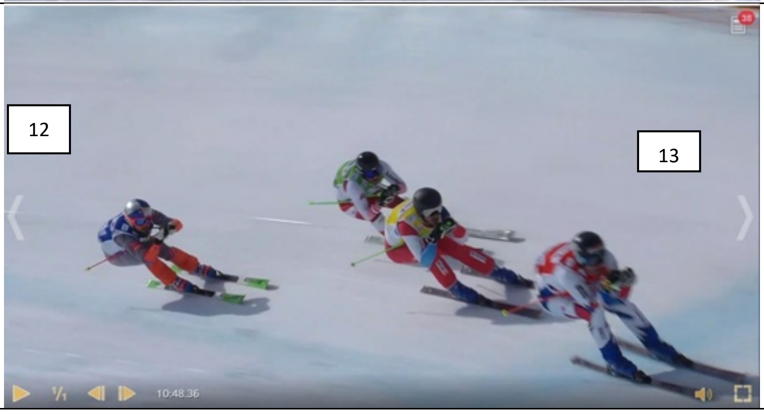		12= Bank 2 entry<br>13= Bank 2 exit<br>(Exit is marked with the blue line)

5	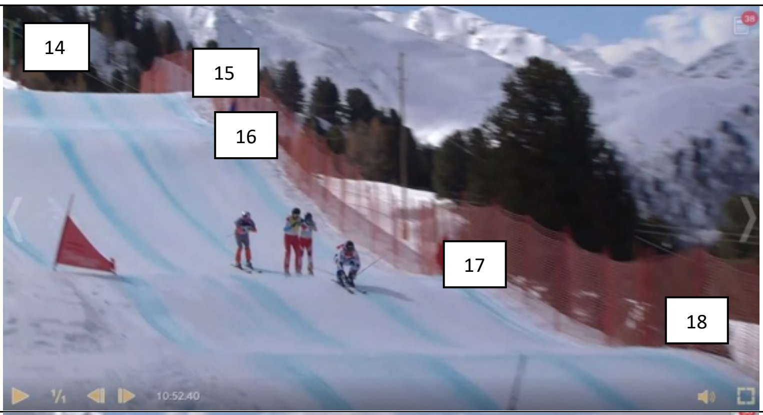		14= Dragon up<br>15= Dragon peak<br>16= Dragon down<br>17= Roller<br>18= Roller
6	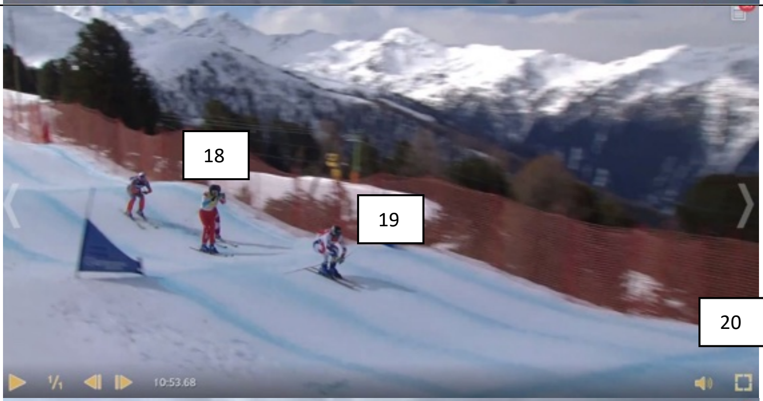 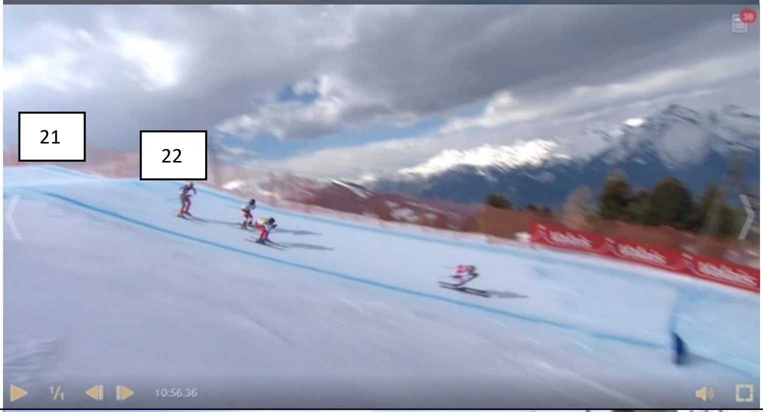		19= Roller<br>20= Roller<br>21= Roller<br>22= Roller
7	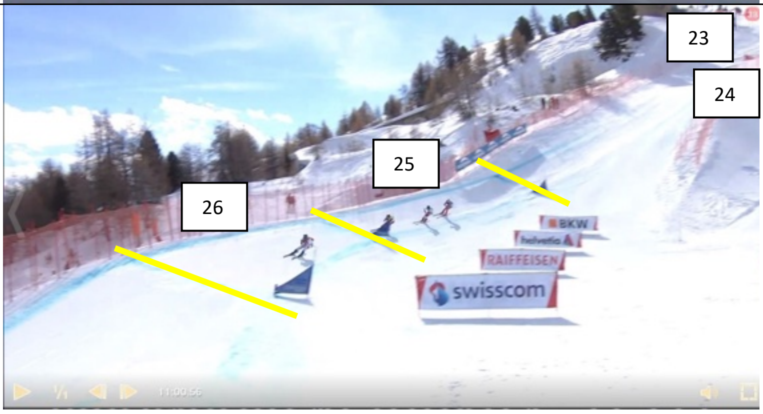		23= Roller<br>24= Roller<br>25= Bank 3 entry<br>26= Bank 3 exit

	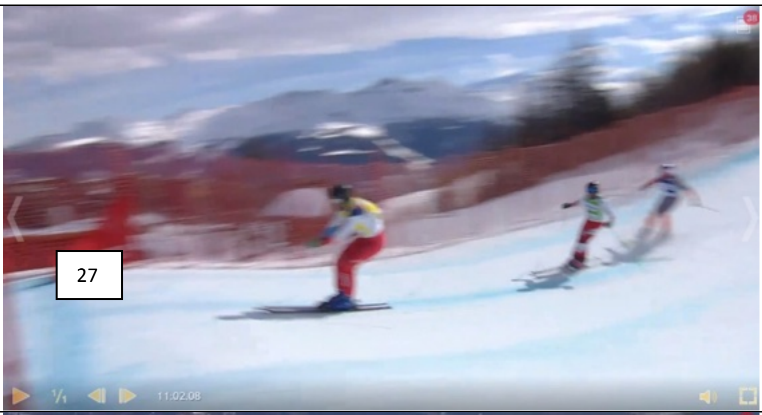		27=Roller
8	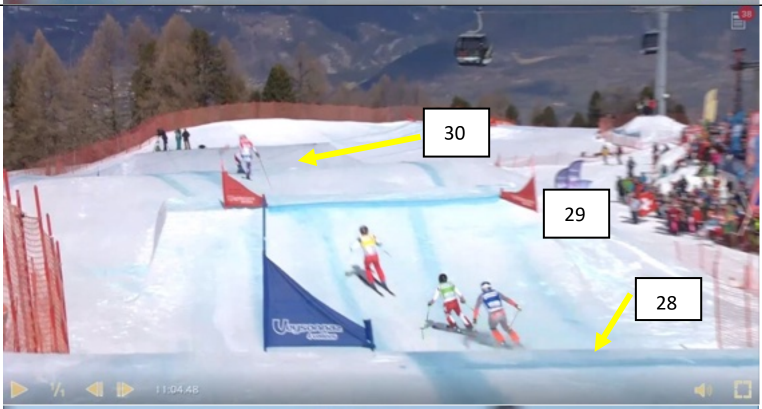<br>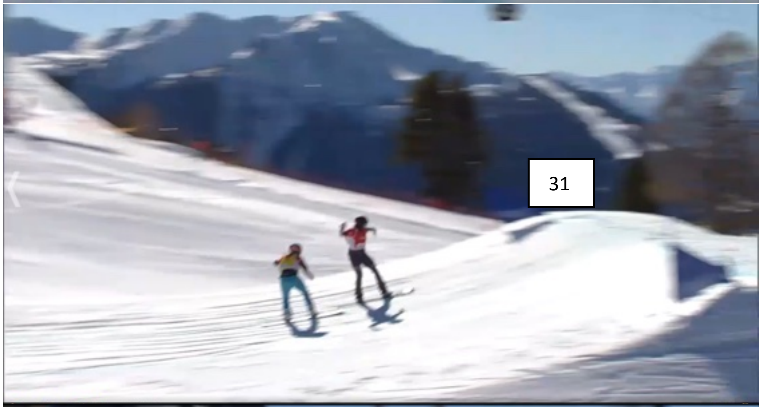<br>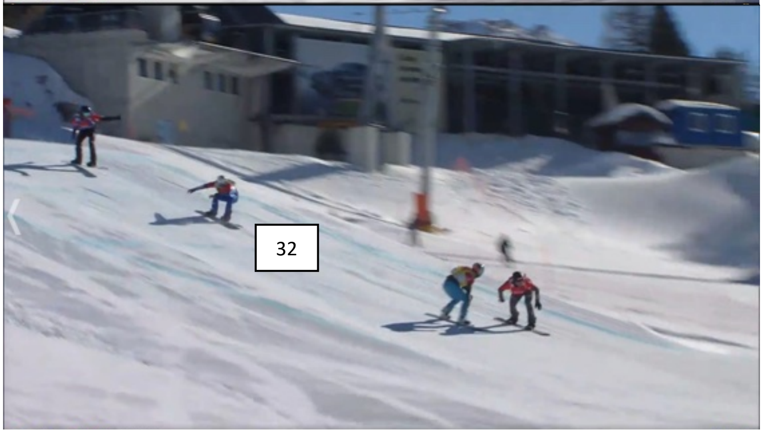		**SBX:**<br>28= Roller<br>29= Major jump 3<br>30= Landing<br><br>31= Major Jump 4<br><br>32= Landing

<table>
<tr>
<td></td>
<td>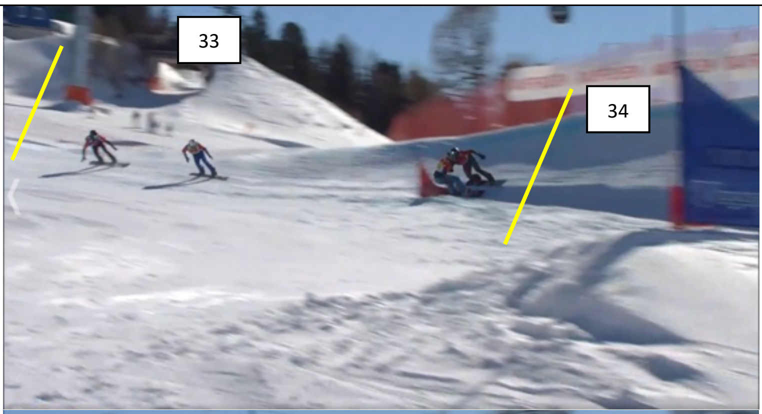

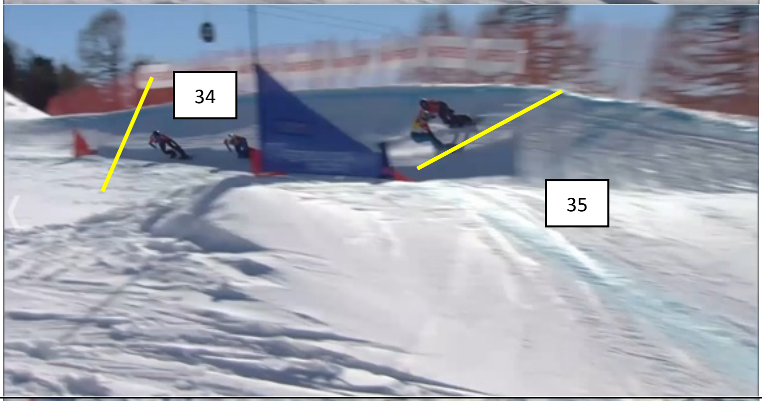
</td>
<td></td>
<td>33= Bank 4 entry
34= Bank 4 exit

35=Roller</td>
</tr>
<tr>
<td>9</td>
<td>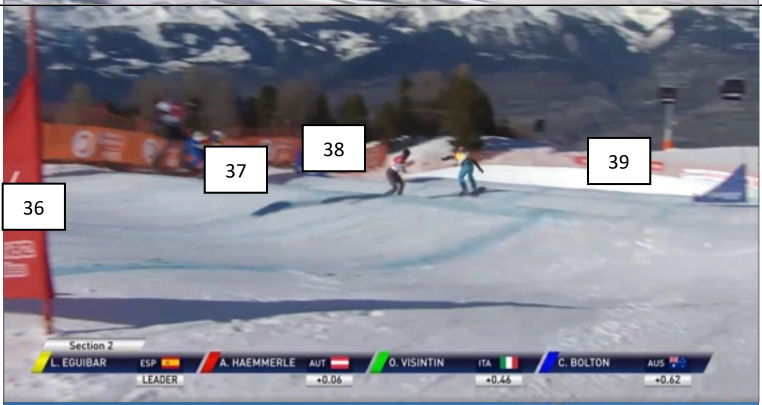

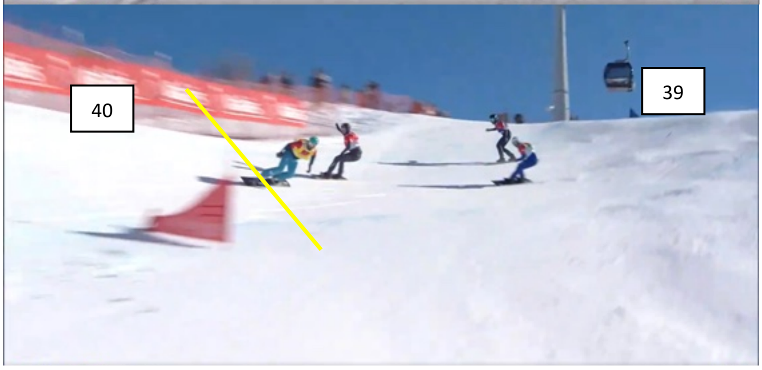
</td>
<td></td>
<td>36= Roller
37= Roller
38= Roller
39= Roller

40=Bank 5 entry</td>
</tr>
</table>

<table>
<tr><td></td><td>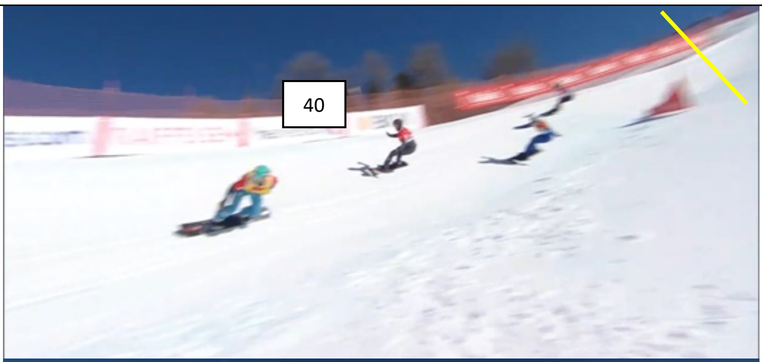

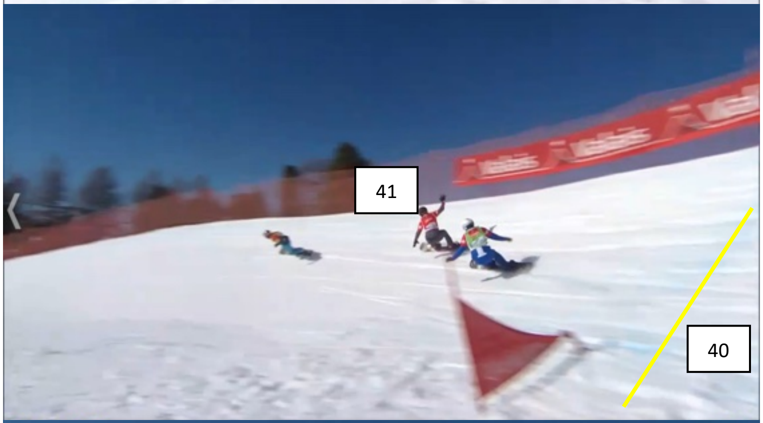

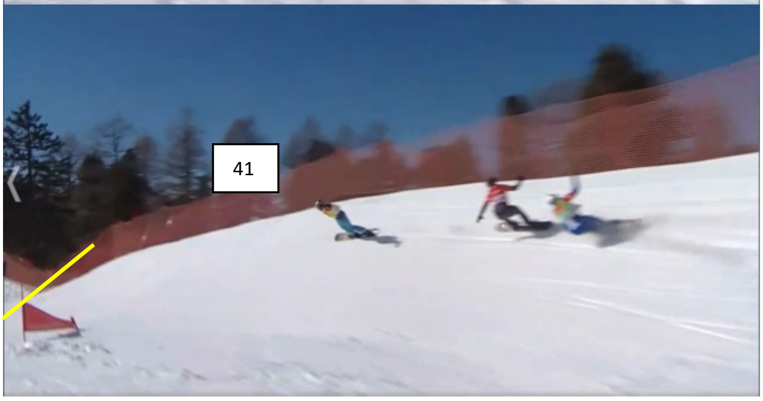
</td><td></td><td>41=Bank 5 exit</td></tr>
<tr><td>10</td><td>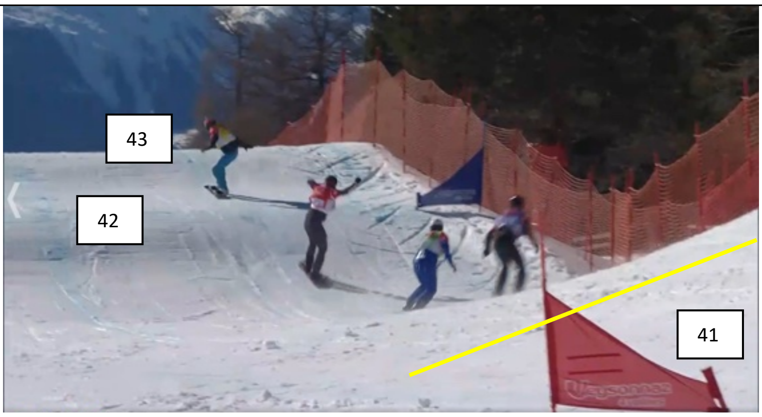
</td><td></td><td>42= Pyramid up
43= Pyramid</td></tr>
</table>

	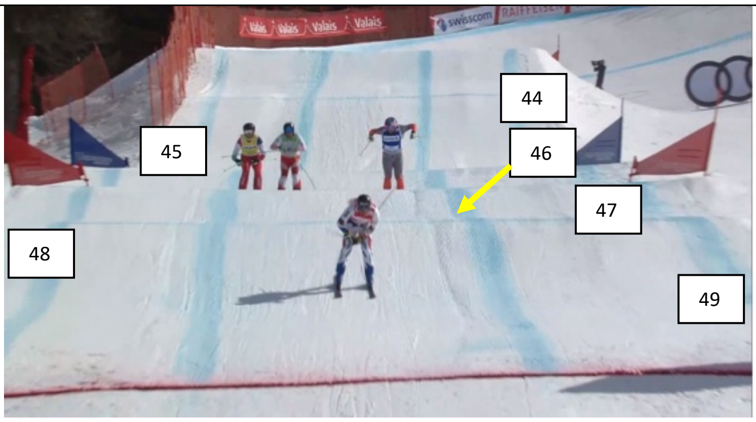		44= Pyramid down<br>45= Roller<br>46= Roller<br>47= Roller<br>48= Roller<br>49= Finish line

Segment	***Obstacle***	***SX***
1	*1*	*Starttable*
	2	*Startdrop*
	3	*Wu tang 1*
	4	*Bathtub, entry*
	5	*Bathtub, exit*
	6	*Major Jump1*
2	*7*	*Landing*
	8	*Major Jump2*
3	*9*	*Landing*
	10	*Bank 1 entry*
	11	*Bank 1 exit*
4	*12*	*Bank 2 entry*
	13	*Bank 2 exit*
5	*14*	*Dragon up*
	15	*Dragon peak*
	16	*Dragon down*
	17	*Roller*
	18	*Roller*
6	*19*	*Roller*
	20	*Roller*
	21	*Roller*
	22	*Roller*
7	*23*	*Roller*
	24	*Roller*
	25	*Bank 3 entry*
	26	*Bank 3 exit*
	27	*Roller*
	28	*Roller*
8	*29*	*Major jump 3*
	30	*Landing*
	31	*Major jump 4*
	32	*Landing*
	33	*Bank 4 entry*
	34	*Bank 4 exit*
9	*35*	*Roller*
	36	*Roller*
	37	*Roller*
	38	*Roller*
	39	*Roller*
	40	*Bank 5 entry*
	41	*Bank 5 exit*
10	*42*	*Pyramid up*
	43	*Pyramid*
	44	*Pyramid down*
	45	*Roller*
	46	*Roller*
	47	*Roller*
	48	*Roller*
	49	*Finsih line*

Idre Track Information

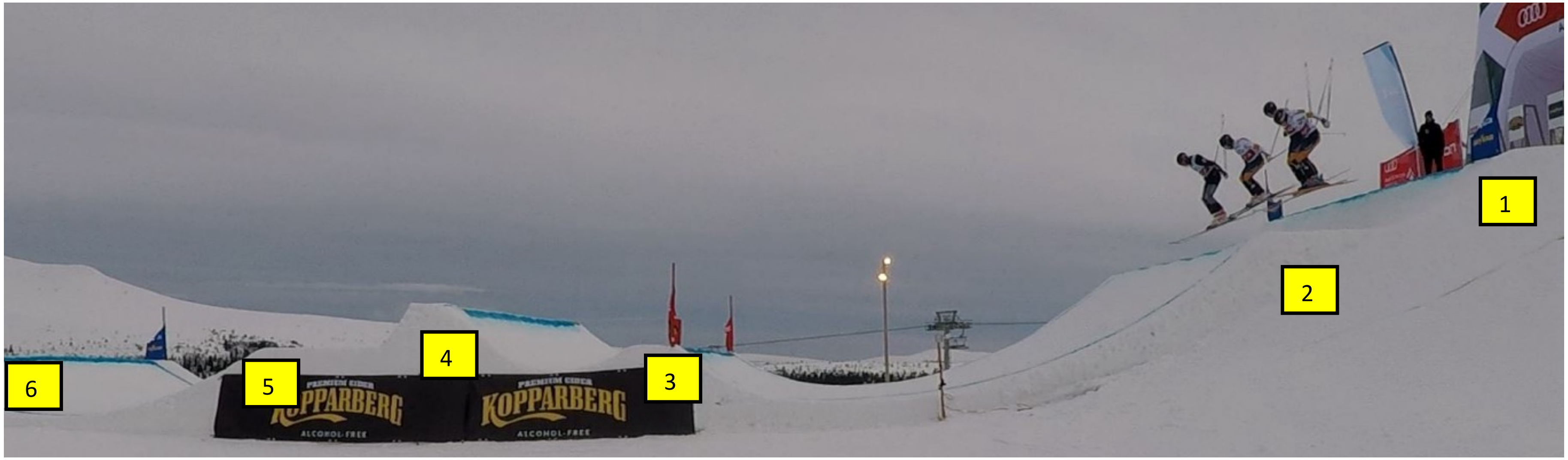

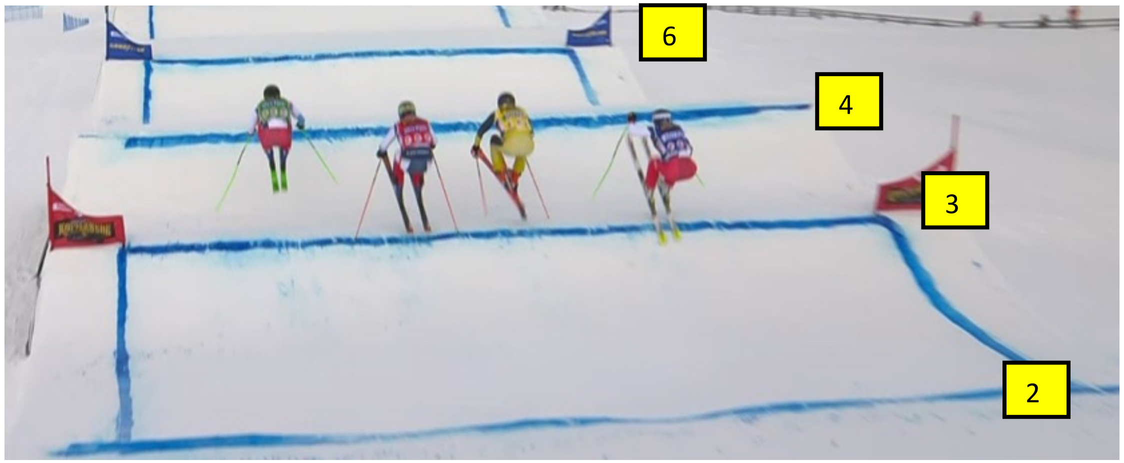

Segment	***Obstacle***	***Description***
1	*1*	*Starttable*
	2	*Startdrop*
	3	*Stepup up*
	4	*Wu tang*
	5	*Landing*
	6	*Wu tang*

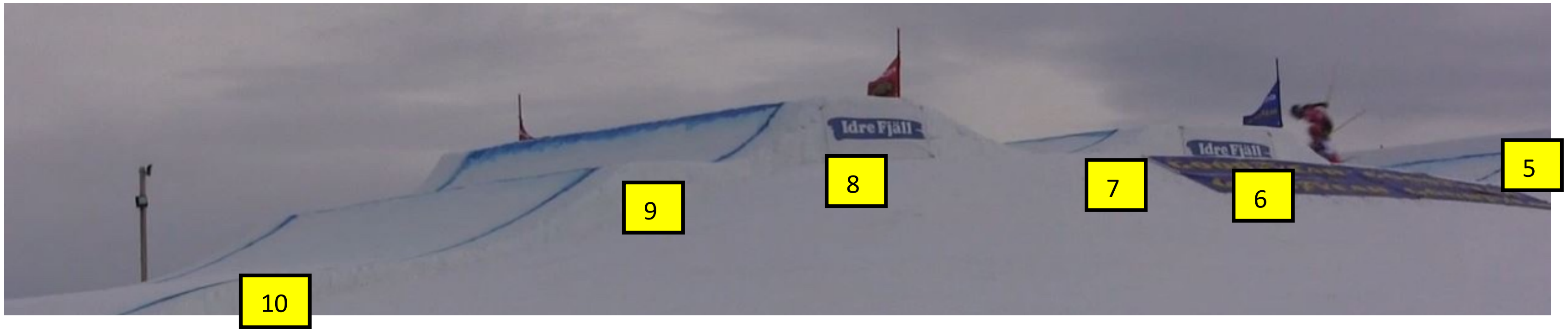

Segment	***Obstacle***	***Description***	***Obstacle***	***Description***
1	*7*	*Landing*	*13*	*Roller 3*
	8	*Wu tang*		
	9	*Landing*		
	10	*Roller*		
	11	*Roller1*		
	12	*Roller2*		

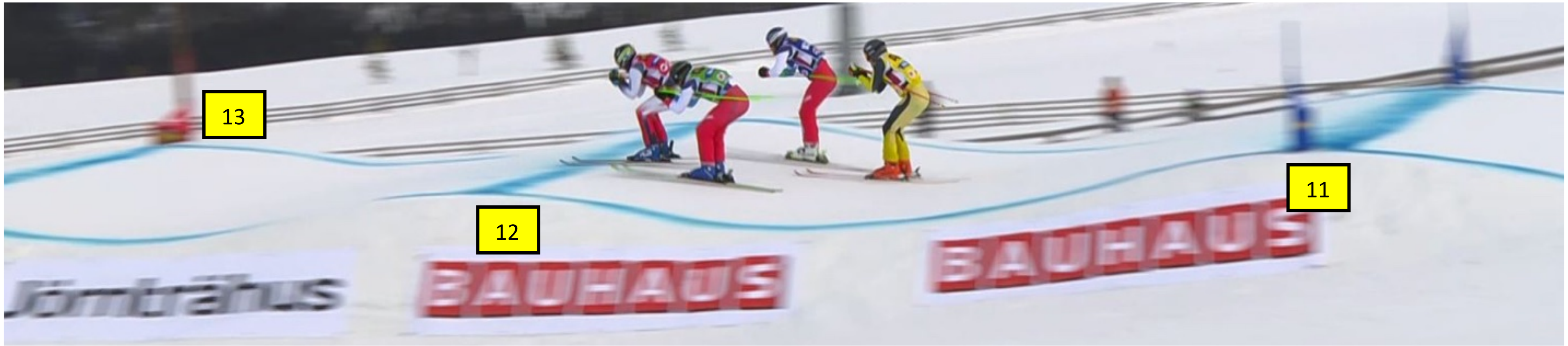

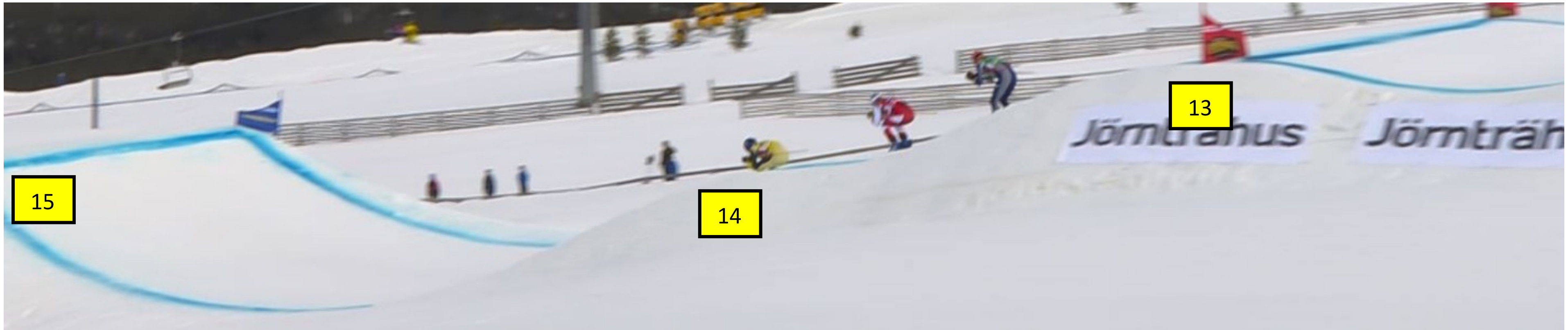

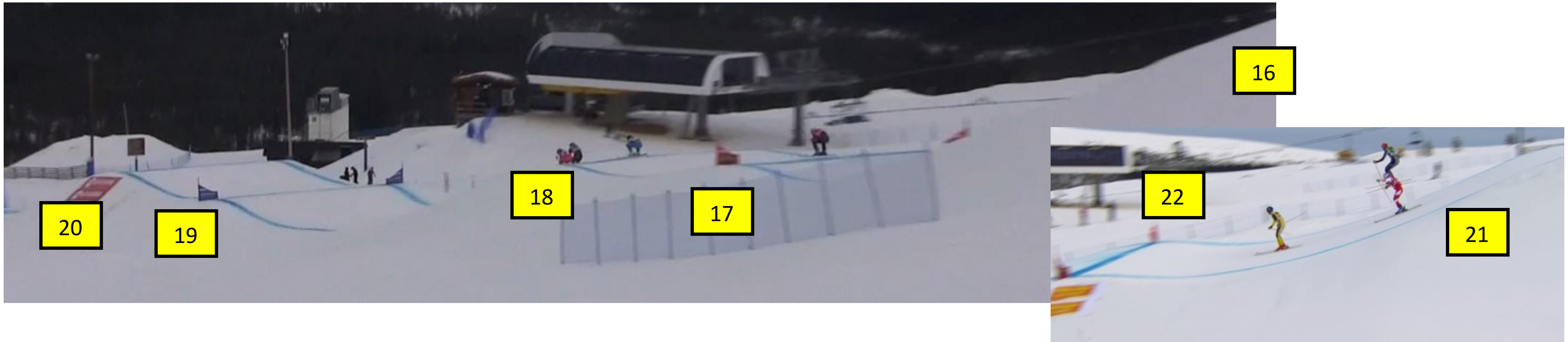

Segment	***Obstacle***	***Description***	***Obstacle***	***Description***
1	*14*	*Roller*	*20*	*Step up second*
	15	*Major Jump take off*	*21*	*Landing*
	16	*Landing*	*22*	*Roller*
	17	*Double first*	*23*	*Roller*
	18	*Double second*		
	19	*Step up first*		

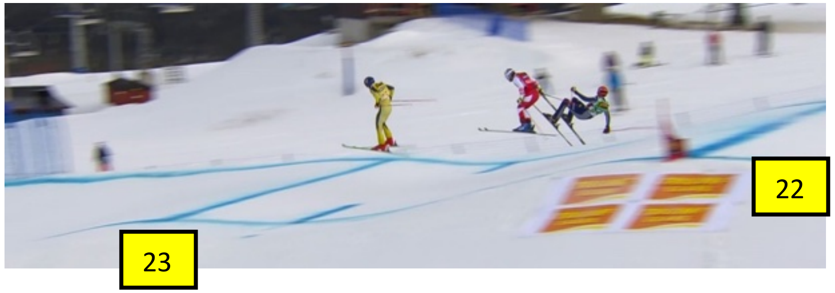

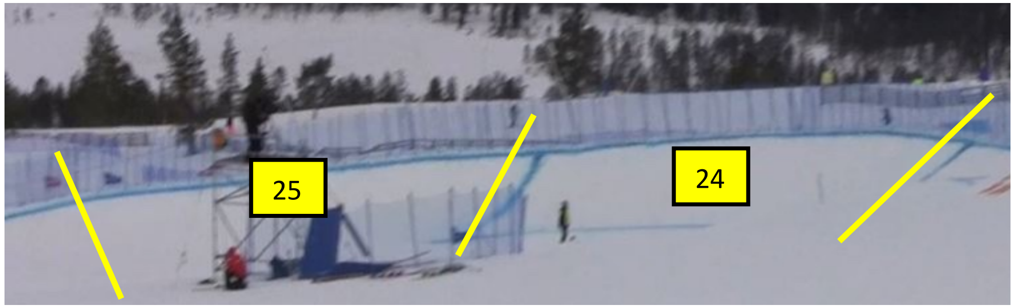

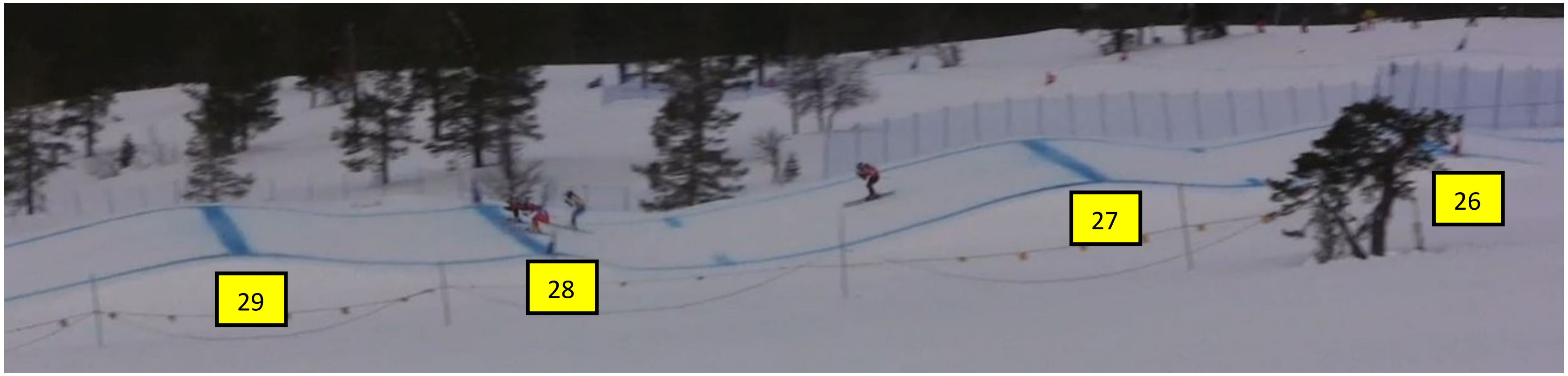

Segment	*Obstacle*	*Description*
2	*24*	*Bank entry*
	25	*Bank exit*
	26	*Roller*
	27	*Roller*
	28	*Roller*
	29	*Roller*

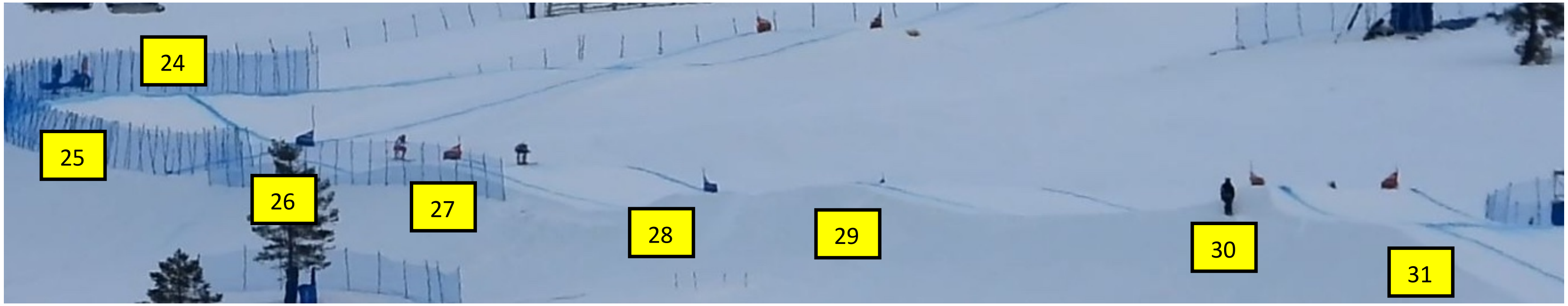

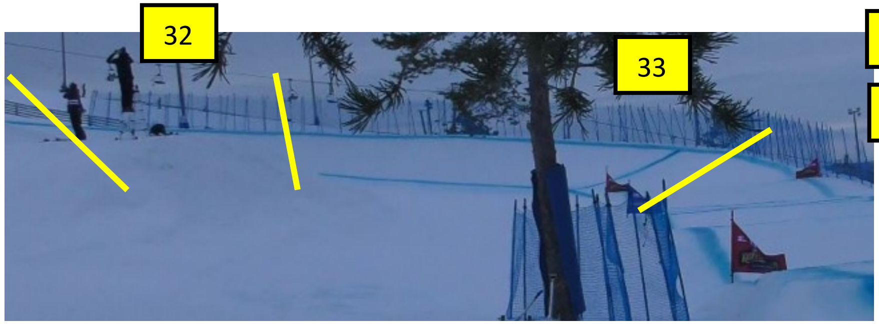

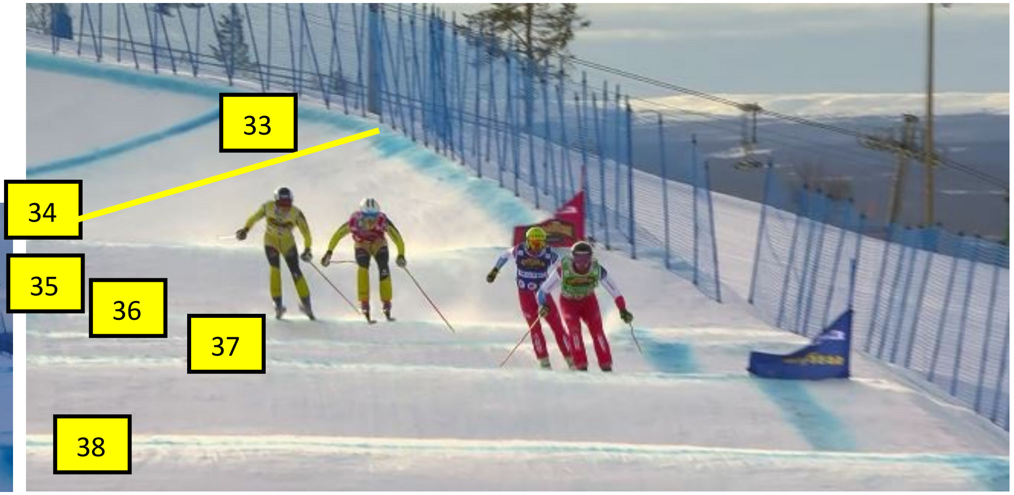

Segment	***Obstacle***	***Description***	***Segment***	***Obstacle***	***Description***	***Obstacle***	***Description***
2	*24*	*Bank entry*		*30*	*Jump take off*	*36*	*Roller*
	25	*Bank exit*		*31*	*Landing Jump*	*37*	*Roller*
	26	*Roller*	***3***	*32*	*Bank turn entry (starts directly at landing 31)*	*38*	*Roller*
	27	*Roller*		*33*	*Bank turn exit*		
	28	*Roller*		*34*	*Roller*		
	29	*Roller*		*35*	*Roller*		

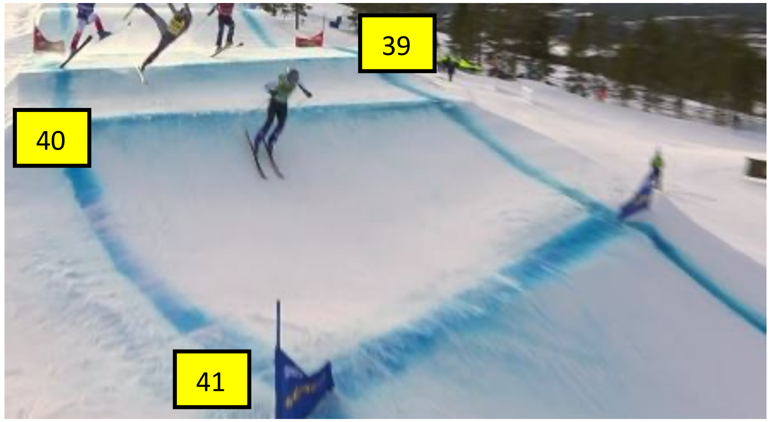

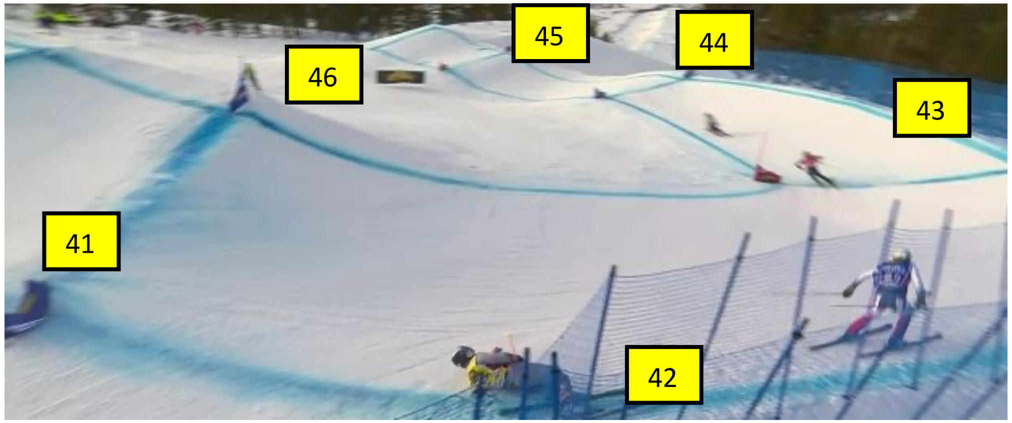

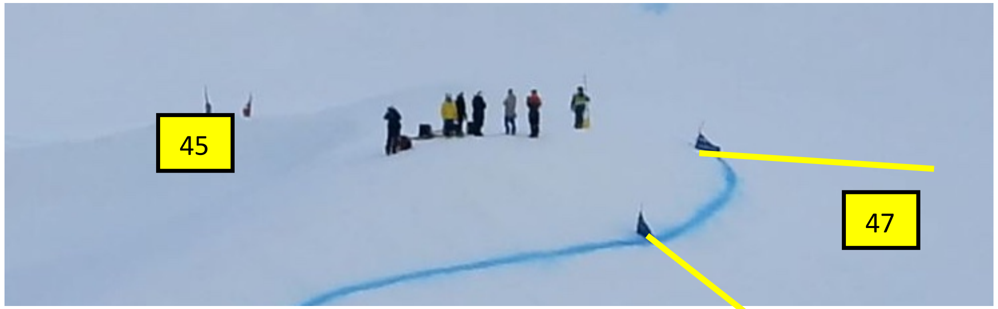

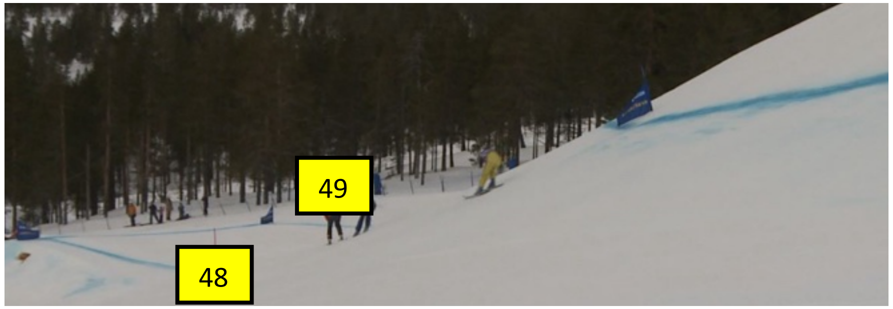

Segment	*Obstacle*	*Description*	*Segment*	*Obstacle*	*Description*	*Segment*	*Obstacle*	*Description*
3	*39*	*Wu tang*		*44*	*Roller*	***5***	*49*	*Roller*
	40	*Landing*		*45*	*Roller*			
	41	*corner*		*46*	*Roller*			
4	*42*	*Bank turn entry*		*47*	*Negative turn*			
	43	*Bank turn exit*		*48*	*GS Gate*			

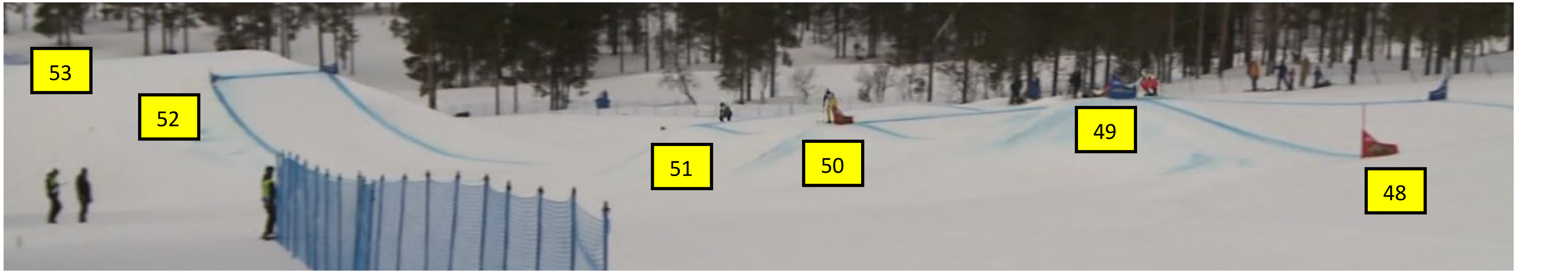

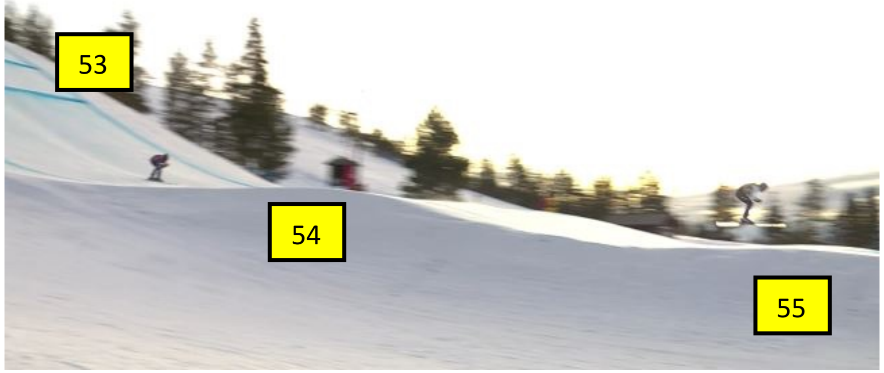

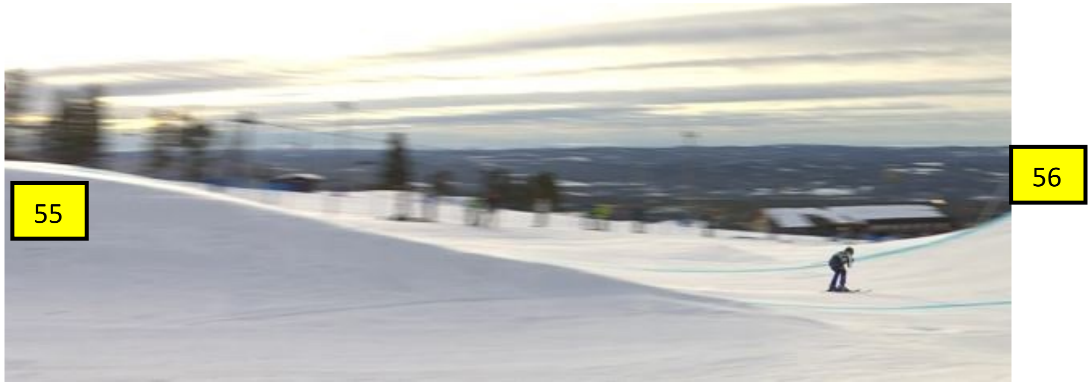

Segment	***Obstacle***	***Description***	***Segment***	***Obstacle***	***Description***
5	*50*	*Roller*	***6***	*55*	*Roller*
	51	*Roller*		*56*	*Jump take off*
6	*52*	*Jump take off*			
	53	*Jump landing*			
	54	*Roller*			

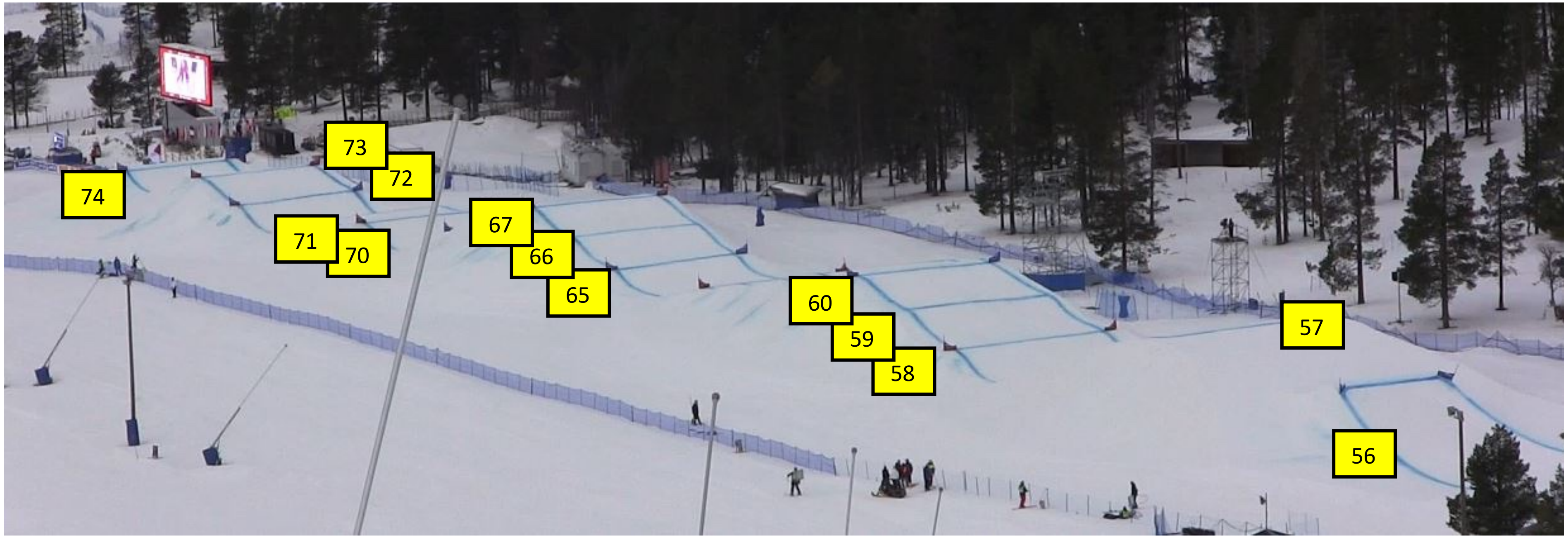

Segment	*Obstacle*	*Description*	*Obstacle*	*Description*	*Obstacle*	*Description*	*Obstacle*	*Description*
6	*57*	*Jump landing*	*63*	*Dragon down3*	*69*	*Dragon down2*	*75*	*Kicker landing*
	58	*Dragon up1*	*64*	*Dragon down4*	*70*	*Double first*		
	59	*Dragon up2*	*65*	*Dragon up1*	*71*	*Double second*		
	60	*Dragon peak*	*66*	*Dragon up2*	*72*	*Step up1*		
	61	*Dragon down1*	*67*	*Dragon peak*	*73*	*Step up2*		
	62	*Dragon down2*	*68*	*Dragon down1*	*74*	*Kicker take off*		

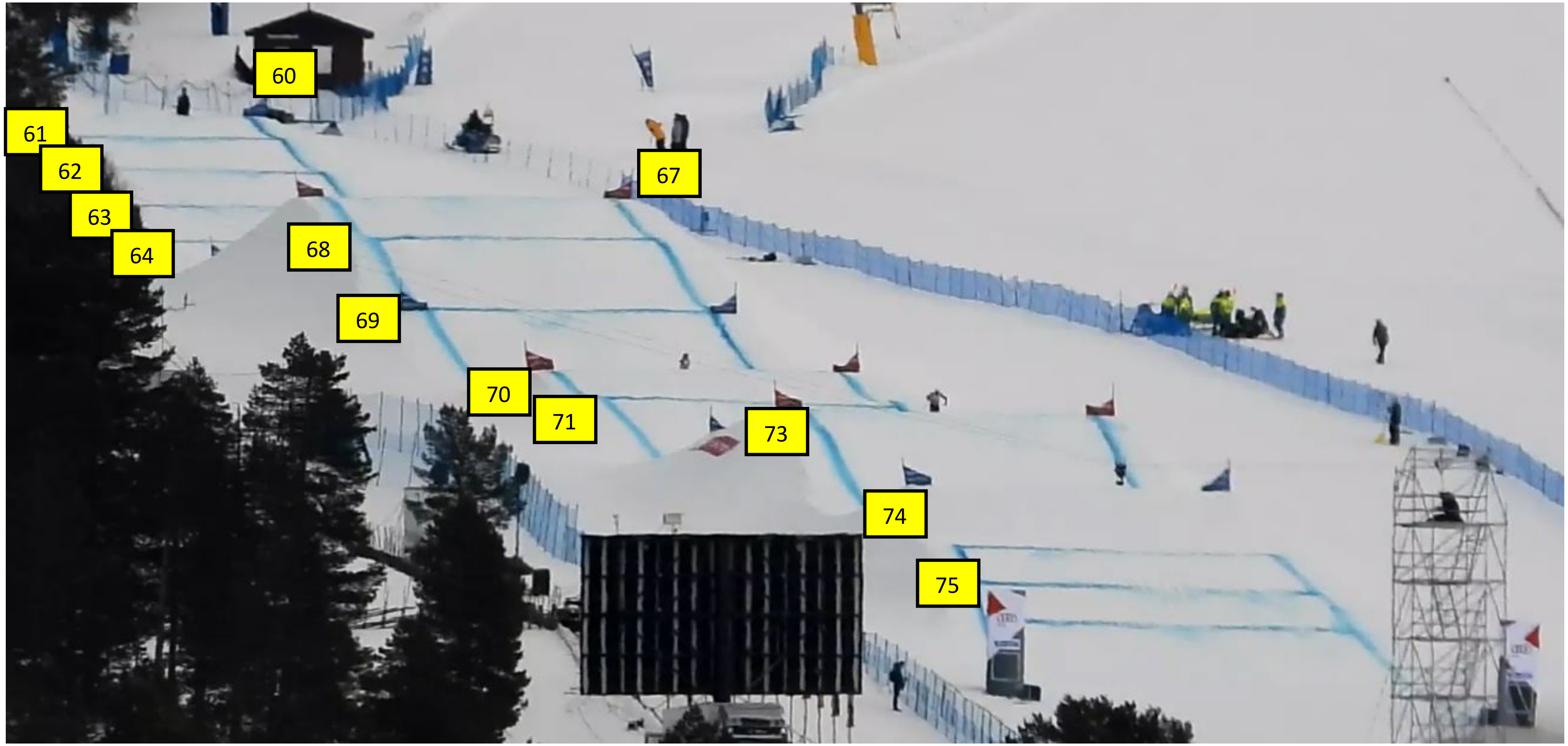

Segment	Obstacle	Description	Obstacle	Description	Obstacle	Description	Obstacle	Description
**6**	57	Jump landing	63	Dragon down3	69	Dragon down2	75	Kicker landing
	58	Dragon up1	64	Dragon down4	70	Double first	76	After finish line
	59	Dragon up2	65	Dragon up1	71	Double second		
	60	Dragon peak	66	Dragon up2	72	Step up1		
	61	Dragon down1	67	Dragon peak	73	Step up2		
	62	Dragon down2	68	Dragon down1	74	Kicker take off		

Innichen Track Information

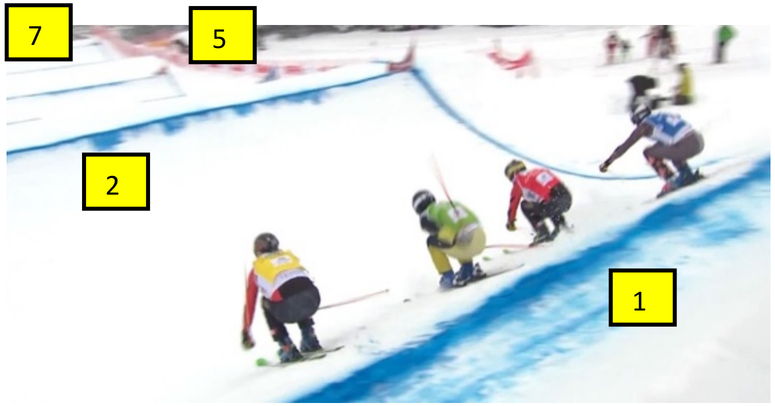

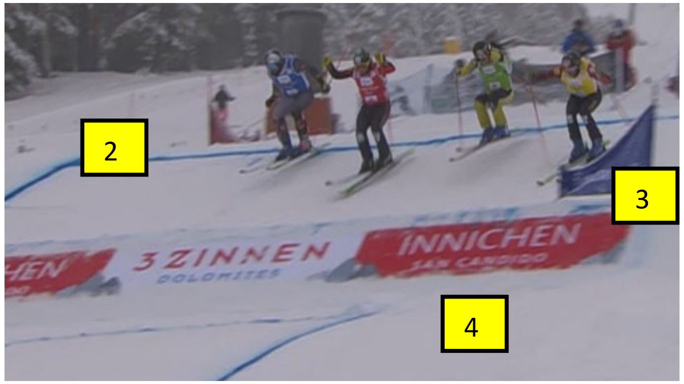

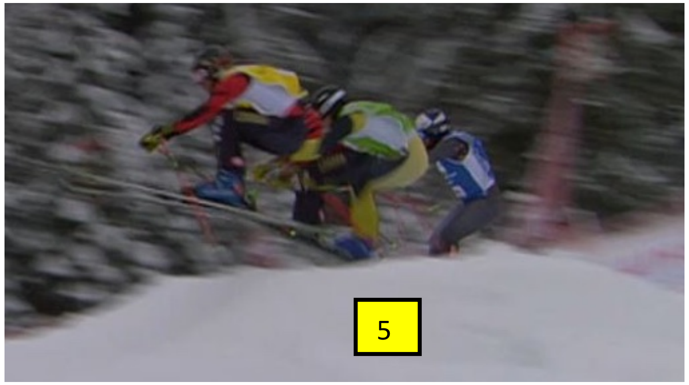

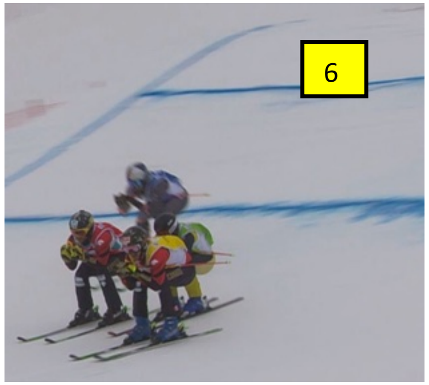

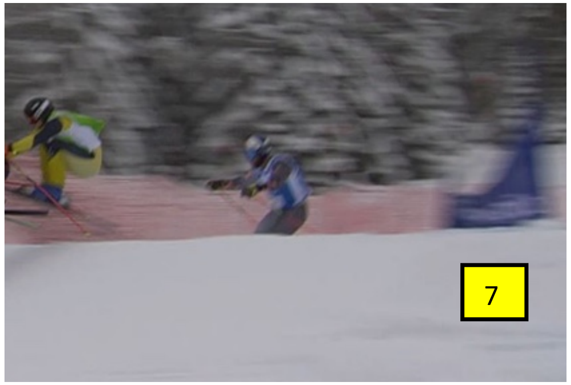

Segment	*Obstacle*	*Description*	*Obstacle*	*Description*
1	*1*	*Startjump*	*8*	*Landing Wu tang 3*
	2	*Wu tang 1*		
	3	*Jump 3 Zinnen*		
	4	*Landing 3 Zinnen*		
	5	*Wu tang2*		
	6	*Landing 2*		
	7	*Wu tang 3*		

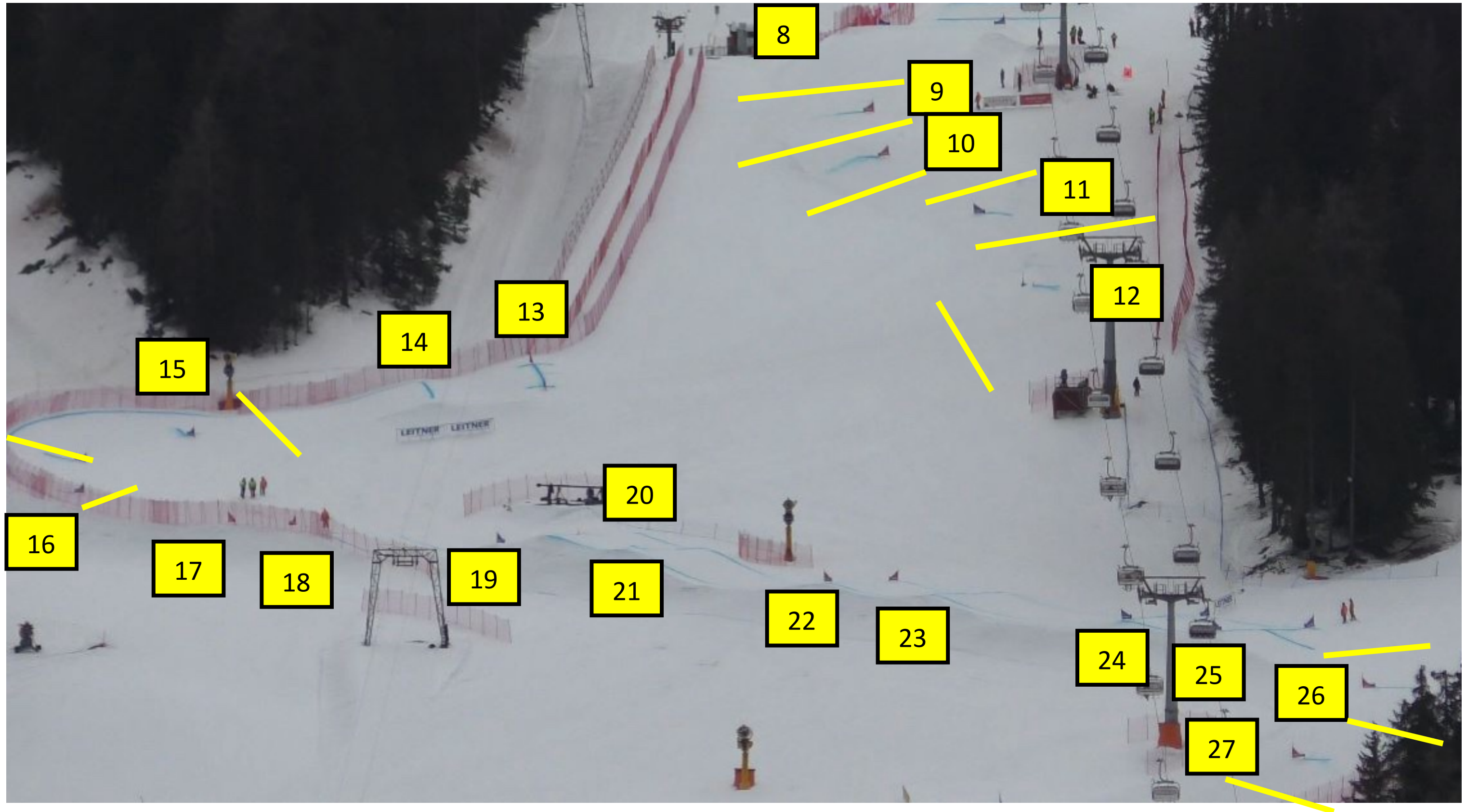

Segment	***Obstacle***	***Description***	***Obstacle***	***Description***	***Obstacle***	***Description***	***Obstacle***	***Description***
2	*9*	*Turn1 entry + Roller*	*15*	*Bank1 entry*	*21*	*Triple down*	*27*	*Negative exit*
	10	*Turn1 exit*	*16*	*Bank1 exit*	*22*	*Double first*		
	11	*Turn2 entry + Roller*	*17*	*Roller3*	*23*	*Double second*		
	12	*Turn2 exit*	*18*	*Roller4*	*24*	*Double first*		
	13	*Roller1*	*19*	*Triple up*	*25*	*Double second*		
	14	*Roller2*	*20*	*Triple peak*	*26*	*Negative entry*		

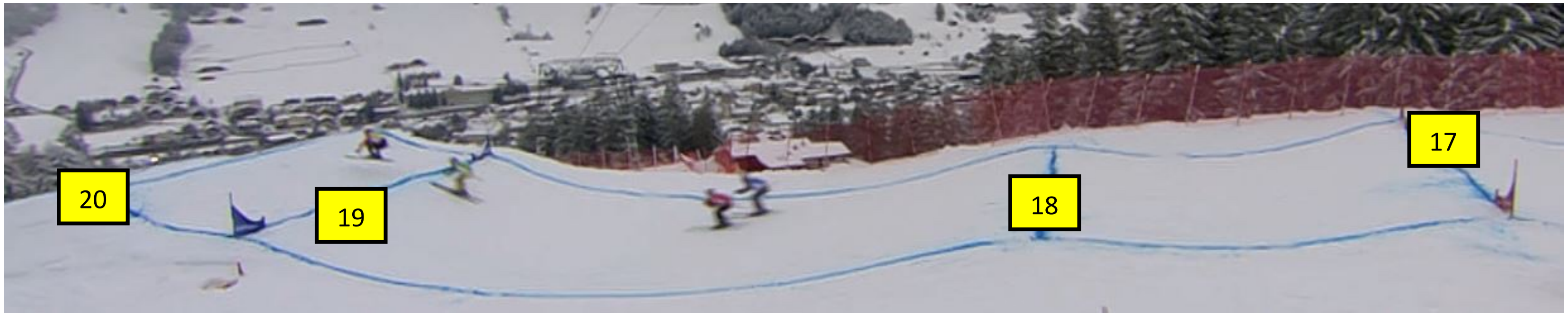

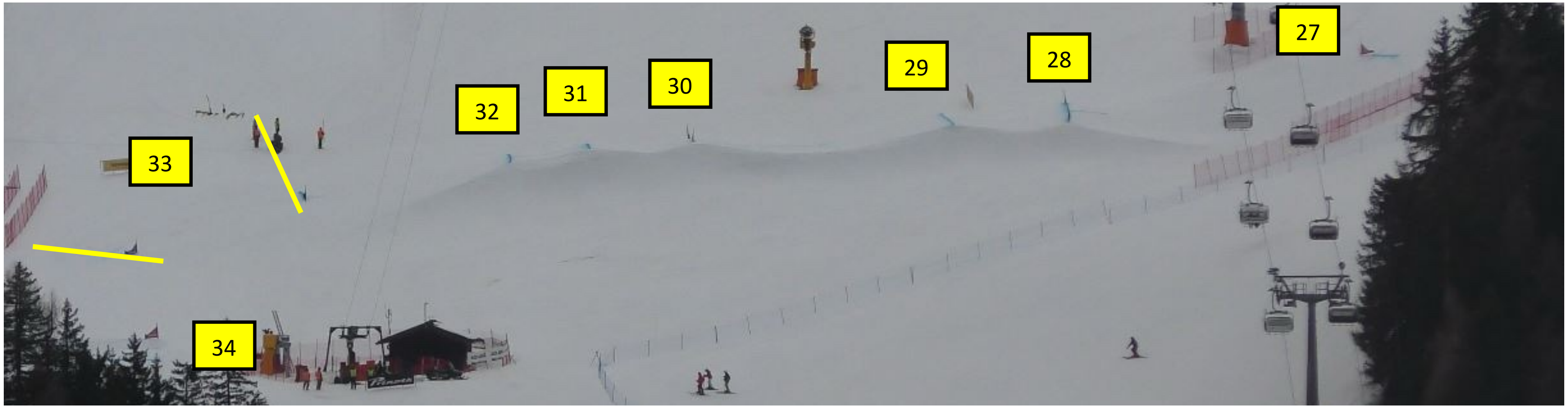

Segment	***Obstacle***	***Description***	***Obstacle***	***Description***
3	*28*	*Double first*	*33*	*GS Turn*
	29	*Double second*	*34*	*Jump take off*
	30	*Triple first*		
	31	*Triple second*		
	32	*Triple third*		
	14	*Roller2*		

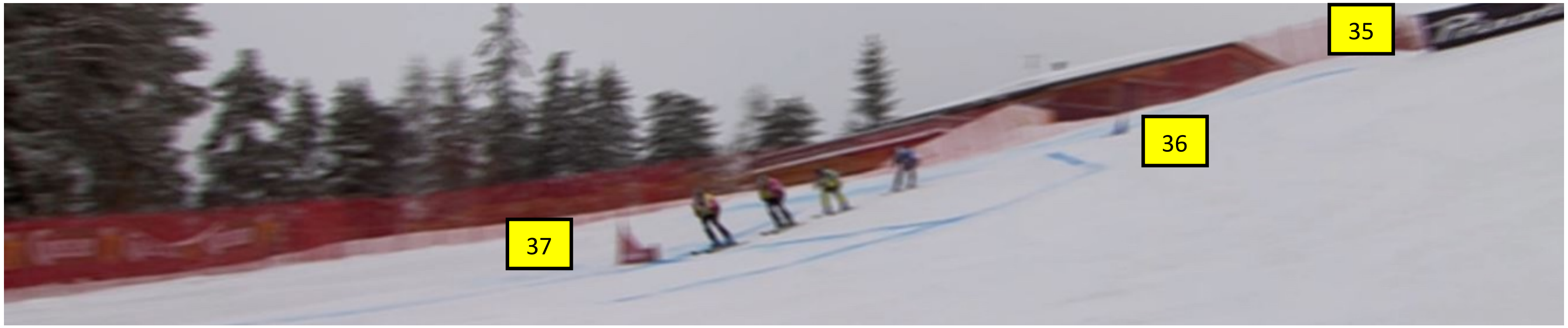

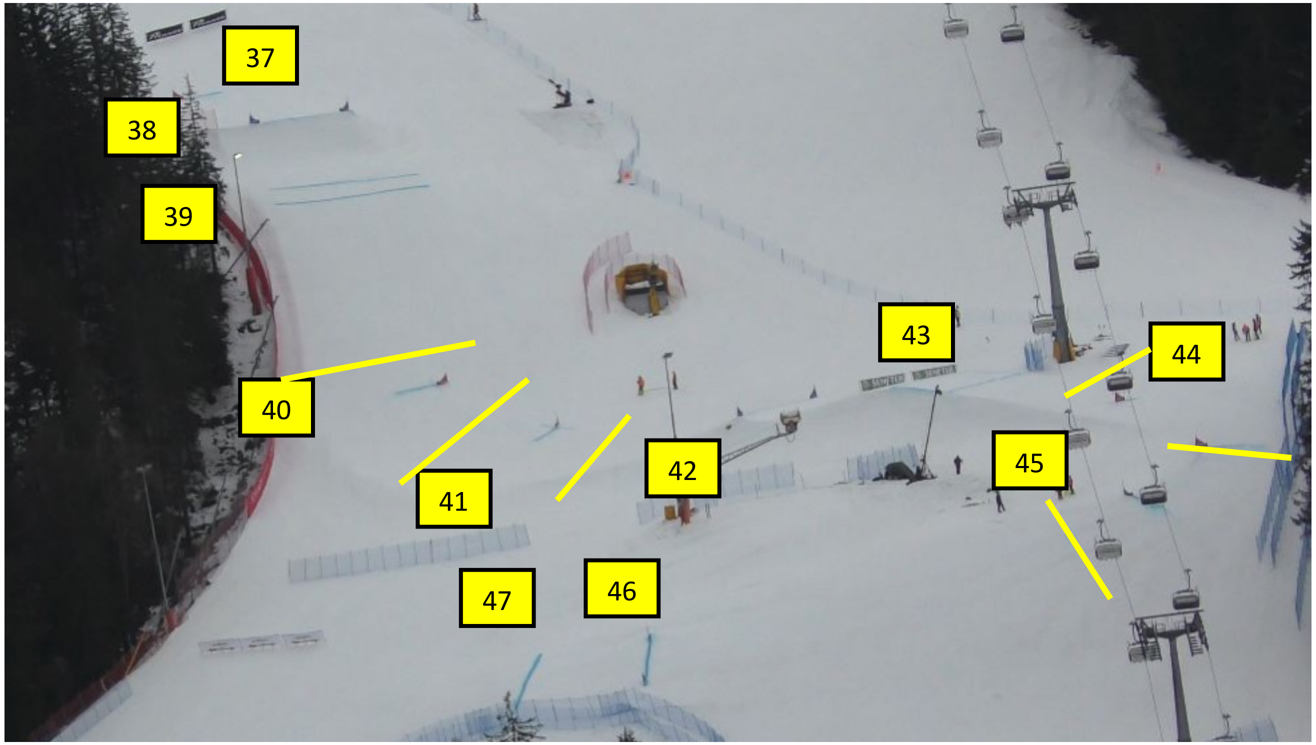

Segment	***Obstacle***	***Description***
4	*35*	*Landing*
	36	*GS Gate*
	37	*GS Gate*
	38	*Jump take off*
	39	*Landing*
	40	*Compression turn entry*
	41	*Compression turn exit*
	42	*Step up 1*
	43	*Step up 2*
	44	*Big negative entry*
	45	*Big negative exit*
	46	*Roller*
	47	*Roller*

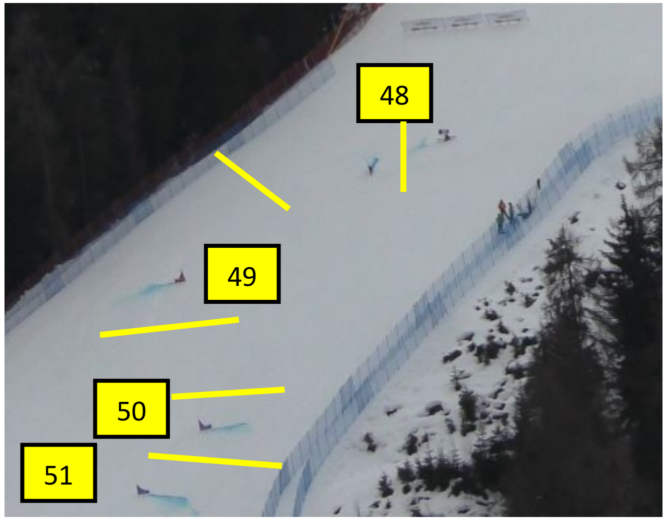

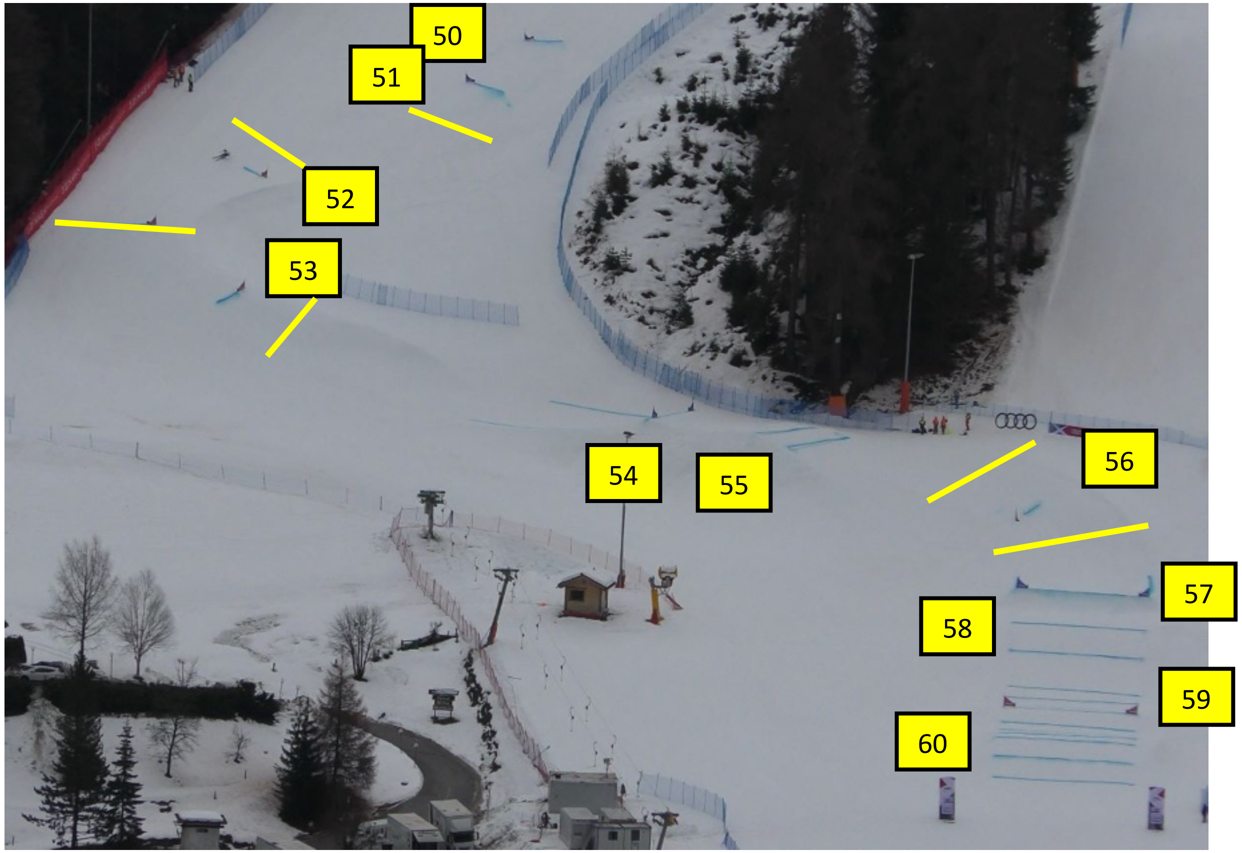

Segment	***Obstacle***	***Description***	***Obstacle***	***Description***	***Obstacle***	***Description***
5	*48*	*Turn entry*	*54*	*Kicker take off*	*60*	*Kicker landing*
	49	*Turn exit*	*55*	*Kicker landing*		
	50	*Turn entry*	*56*	*Bank turn*		
	51	*Turn exit*	*57*	*Kicker take off*		
	52	*Negative turn entry*	*58*	*Kicker landing*		
	53	*Negative turn exit*	*59*	*Kicker take off*		
